# ADAR1 Controls Macrophage Scavenging and Lipid‐Buffering Programs in Metabolic Tissues

**DOI:** 10.1002/eji.70189

**Published:** 2026-04-26

**Authors:** Achilleas Fardellas, Emelie Barreby, Madara Brice, Sebastian Nock, Jules Russick, Ana Vankova, Charlotte Edberg, Ida Robertsen, Jens K. Hertel, Per Stål, Gunnar Mellgren, Cecilia Karlsson, Jøran S. Hjelmesaeth, Hannes Hagström, Erik Näslund, Volker M. Lauschke, Johan Fernø, Ping Chen, Cecilia Morgantini, Myriam Aouadi, Niklas K. Björkström

**Affiliations:** ^1^ Department of Medicine, Huddinge Center for Infectious Medicine Karolinska Institutet Karolinska University Hospital Stockholm Sweden; ^2^ Division of Surgery Department of Clinical Sciences Danderyd Hospital, Karolinska Institutet Stockholm Sweden; ^3^ Section for Pharmacology and Pharmaceutical Biosciences Department of Pharmacy University of Oslo Oslo Norway; ^4^ Department of Endocrinology Obesity and Nutrition Vestfold Hospital Trust Tønsberg Norway; ^5^ Division of Hepatology Department of Upper GI Diseases Karolinska University Hospital Stockholm Sweden; ^6^ Department of Medicine, Huddinge Karolinska Institutet Karolinska University Hospital Stockholm Sweden; ^7^ Mohn Research Center For Diabetes Precision Medicine Department of Clinical Science University of Bergen Bergen Norway; ^8^ Hormone Laboratory Department of Medical Biochemistry and Pharmacology Haukeland University Hospital Bergen Norway; ^9^ Department of Molecular and Clinical Medicine Institute of Medicine Sahlgrenska Academy at the University of Gothenburg Gothenburg Sweden; ^10^ Late‐stage Development, Cardiovascular, Renal and Metabolism BioPharmaceuticals R&D, AstraZeneca Gothenburg Sweden; ^11^ Department of Endocrinology Morbid Obesity and Preventive Medicine Institute of Clinical Medicine University of Oslo Oslo Norway; ^12^ Department of Physiology and Pharmacology and Center for Molecular Medicine Karolinska Institutet Stockholm Sweden; ^13^ Dr Margarete Fischer‐Bosch Institute of Clinical Pharmacology Stuttgart Germany; ^14^ University of Tübingen Tübingen Germany; ^15^ Department of Pharmacy the Second Xiangya Hospital Central South University Changsha China; ^16^ Department of Laboratory Medicine Karolinska Institutet Stockholm Sweden; ^17^ Stem Cell and Metabolism Research Program Research Programs Unit University of Helsinki Helsinki Finland; ^18^ Cardio Metabolic Unit Department of Medicine, Huddinge Karolinska Institutet Karolinska University Hospital Stockholm Sweden

## Abstract

Adenosine deaminase acting on RNA 1 (ADAR1) regulates mRNA fate and function through adenosine‐to‐inosine (A‐to‐I) RNA editing and RNA‐binding activities. While its role in innate immunity is established, the broader regulatory functions of ADAR1 in macrophages remain poorly defined. Here, we systematically profiled ADAR1 expression across human immune cells and identified marked enrichment in macrophages, driven by selective usage of an alternative transcription start site during monocyte‐to‐macrophage differentiation. ADAR1 binds, edits, and modulates key macrophage targets involved in efferocytosis, endocytosis, lysosomal processing, lipid metabolism, and proliferation in an isoform‐specific manner. We further demonstrate that ADAR1 levels and activity are dynamically regulated in adipose tissue and liver during the progression of metabolic disease. Linked to this, macrophage‐specific ablation of ADAR1 co‐cultured in organotypic 3D primary human liver spheroids and exposed to metabolic stress resulted in an exacerbated lipid accumulation phenotype. Finally, we identify a lipid‐associated macrophage‐specific upregulation of ADAR1 in adipose tissue following weight loss interventions, mechanistically driven by free fatty acids. These findings uncover a previously unrecognized role for ADAR1 in lipid‐buffering, scavenging, and proliferative macrophage functions, extending its biological relevance beyond canonical interferon‐mediated immunity and establishing ADAR1 as a key regulator of macrophage adaptation in metabolic disease.

## Graphical abstract

ADAR1 expression increase during monocyte‐to‐macrophage differentiation via an alternative transcription start site. Isoform‐specific ADAR1 activity governs key macrophage functions, including efferocytosis, endocytosis, lipid metabolism, and proliferation. Moreover, ADAR1 in macrophages responds to different metabolic states and buffers lipotoxicity under high metabolic load. Vectors obtained from https://BioRender.com.

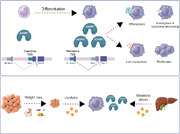

## 1 Introduction

Macrophages are essential mediators of innate immunity, exhibiting considerable plasticity in response to diverse tissue microenvironments and pathophysiological conditions [1, 2]. While the advent of single‐cell RNA‐sequencing (scRNA‐seq) enabled extensive characterization of macrophage transcriptional programs across human and mouse tissues, such approaches primarily capture steady‐state RNA abundance [3, 4]. Increasing evidence indicates that co‐ and post‐transcriptional mechanisms constitute an additional regulatory layer enabling macrophages to mount rapid, context‐dependent, and finely tuned responses to environmental stimuli [5, 6].

One of the most prevalent and biologically consequential post‐transcriptional RNA modifications is adenosine‐to‐inosine (A‐to‐I), catalyzed by adenosine deaminase acting on RNA (ADAR) within double‐stranded RNAs (dsRNAs) [7, 8]. Among the three mammalian ADAR enzymes, ADAR1 is responsible for over 90% of RNA editing activity and exists in two isoforms: the constitutively expressed nuclear p110 and the interferon‐inducible p150, which localizes to both nucleus and cytoplasm [9, 10, 11]. ADAR1‐mediated A‐to‐I editing can modulate RNA secondary structure, influence RNA‐binding interactions, and drive diverse functional consequences depending on the genomic location [8]. In humans, A‐to‐I editing occurs predominantly in repetitive, noncoding, primate‐specific Alu elements, whose sense‐antisense pairing renders them prone to forming immunogenic self‐dsRNAs [7, 12]. A key function of ADAR1 is to bind and edit these self‐dsRNAs, preventing their recognition by cytoplasmic antiviral immune sensors, thereby suppressing aberrant type I interferon (IFN) activation and maintaining immune homeostasis [13, 14, 15]. Loss of ADAR1 in macrophages confirms its role in preventing IFN response in the context of cancer and infectious disease [16, 17]. Beyond its canonical role in suppressing dsRNA‐driven immunity, however, the broader functional significance of ADAR1 in macrophage biology remains poorly understood.

Emerging evidence suggests that ADAR1 interacts with various microRNAs in murine macrophages through both RNA editing‐dependent and independent mechanisms, with opposing effects depending on the disease context [18, 19, 20, 21]. We have previously demonstrated that A‐to‐I RNA editing in human liver macrophages plays a protective role against hepatic insulin resistance in obesity [22]. Consistent with a role in metabolic adaptation, a recent report found that ADAR1 is selectively upregulated in CD68^+^ liver macrophages in response to high‐fat diet feeding in mice [23]. Together, these observations suggest that ADAR1 may regulate macrophage functions beyond immune suppression, particularly in metabolically stressed tissues where macrophage plasticity is critical for maintaining lipid homeostasis and tissue integrity.

Here, we systematically analyzed ADAR1 expression profiles across immune cells in human peripheral blood and tissues and identified marked enrichment of ADAR1 in macrophages during monocyte‐to‐macrophage differentiation driven by an upstream alternative transcription start site (TSS). Using functional knockdown and rescue experiments in human macrophages, coupled with global analyses of the A‐to‐I RNA editome, transcriptome, and proteome, we uncovered isoform‐specific effects of ADAR1 in regulating fundamental macrophage programs, including efferocytosis, endocytosis, lysosomal processing, lipid metabolism, and proliferation. Interestingly, ADAR1 enrichment was most pronounced in human macrophage subpopulations whose predicted functions aligned with these RNA edited pathways. Integration of publicly available clinical RNA‐seq datasets revealed a dynamic relationship between ADAR1 expression, activity, and metabolic disease progression. Using organotypic 3D primary human hepatic spheroid models incorporating ADAR1‐deficient macrophages, we validated that loss of ADAR1 exacerbates lipid accumulation under metabolic stress. Finally, analysis of a longitudinal weight loss cohort demonstrated that ADAR1 is specifically upregulated in LAMs following weight loss, possibly driven by the local lipolytic milieu.

Together, these findings indicate that ADAR1 modulates prototypical macrophage programs with important implications for macrophage adaptations in metabolic disease and weight loss.

## 2 Results

### 2.1 ADAR1 is Enriched in Human Macrophages Ex Vivo and in Vitro

**FIGURE 1:**
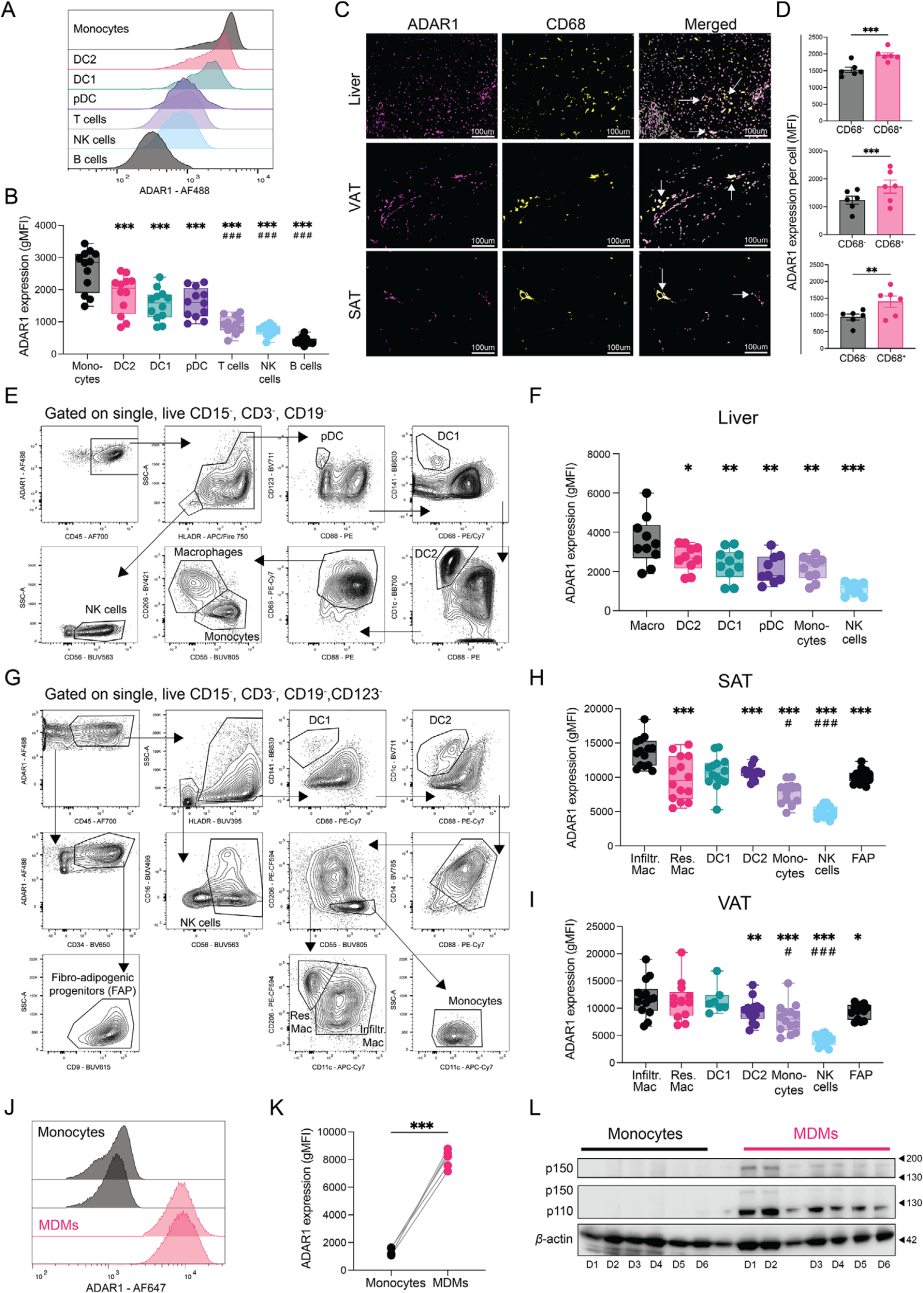
**ADAR1 is enriched in human macrophages ex vivo and in vitro**. (**A, B**) Representative flow cytometry histograms of ADAR1 and summary data (geometric mean fluorescence intensity, gMFI) in the indicated PBMC subpopulations isolated from healthy donor buffy coats (*n* = 12). (**C, D**) Representative 20× immunofluorescence images of FFPE liver, VAT, and SAT biopsies stained for ADAR1 (purple), CD68 (yellow), and counterstained with DAPI (grey), with summary data on ADAR1 MFI in CD68^−^ and CD68^+^ cells (liver, *n* = 6; VAT, *n* = 6; SAT, *n* = 6). MFI was averaged across all cells from 3 to 5 representative images per donor. Scale bar, 100 µm. (**E, F**) Representative flow cytometry gating strategy and summary data for ADAR1 expression in the indicated NPC populations from perfused human livers (*n* = 10). (**G**–**I**) Representative flow cytometry gating strategy and summary data for ADAR1 expression in indicated SVF cell populations from human SAT (*n* = 14) (**H**) and VAT (*n* = 13) **(I**) biopsies in patients with obesity undergoing bariatric surgery. *Displays the comparison to Infiltr. Mac and # the comparison to Res. Mac. (**J**, **K**) Representative histograms and summary data for ADAR1 expression in matched human monocytes and MDMs from healthy donor buffy coats (*n* = 6). (**L**) Western blot analysis of ADAR1 isoforms (p110 and p150) protein expression in matched human monocytes at day 0 and MDMs at day 7 (*n* = 6), with *β*‐actin used as the loading control. Box‐and‐whisker plots display min–max range, interquartile range (25th–75th percentiles), and median. Bar plots show mean ± SEM. Statistical significance was determined by one‐way ANOVA (**B, H**) or mixed‐effects analysis (**F, I**) followed by Tukey's multiple comparisons test, or by paired ratio t‐test (**D, K**). *p < 0.05, **p < 0.01, and ***p < 0.001.

We first investigated ADAR1 expression across a broad spectrum of immune cells in blood and tissues. Analysis of peripheral blood mononuclear cells (PBMCs) using flow cytometry revealed higher ADAR1 expression in monocytes and dendritic cell subsets compared with T cells, NK cells, and B cells (Figure 1A,B; Figure S1A). Among myeloid cells, classical and intermediate monocytes exhibited higher ADAR1 expression than dendritic cells (DCs) (Figure S1B). We then assessed ADAR1 expression in myeloid cells residing in metabolic tissues using immunofluorescence with CD68 as a common myeloid marker. In both human liver and two different adipose tissue depots, visceral (VAT) and abdominal subcutaneous adipose tissues (SAT), CD68^+^ cells exhibited higher ADAR1 fluorescence intensity than nonmyeloid cells (Figure 1C,D). These findings were corroborated by flow cytometry analysis of human liver non‐parenchymal cells (NPCs) and stromal vascular fractions (SVFs) derived from SAT and VAT (Figure 1E–I; Figure S1C,D). In further detail, monocytes, macrophages, and DC subsets exhibited higher ADAR1 expression than NK cells, included as a lymphocyte reference, in both liver and adipose depots (Figure 1F,H,I). In liver tissue, macrophages demonstrated higher ADAR1 expression than monocytes, and a similar pattern was present for infiltrating (Infiltr. Mac) and resident adipose tissue macrophages (Res. Mac) in SAT and VAT (Figure 1F,H,I).

Next, we explored whether the ADAR1 enrichment in macrophages could also be recapitulated in vitro. Upon differentiation using M‐CSF, human monocyte‐derived macrophages (MDMs) exhibited higher ADAR1 expression compared with their corresponding primary monocytes (Figure 1J,K), confirming the results from the ex vivo human tissues. A similar induction was noted using GM‐CSF differentiated macrophages (Figure S2A). To obtain a more quantitative assessment of ADAR1 protein levels, we confirmed ADAR1 induction in MDMs via immunoblot analysis for both p110 and p150 isoforms (Figure 1L), and a subsequent time‐course analysis revealed that ADAR1 increases gradually throughout differentiation (Figure S2B–D). Surprisingly, gene expression analyses revealed no significant differences in *ADAR1* in MDMs and monocytes (Figure S2E), and similar results could be observed when analyzing publicly available datasets (Figure S2F). To investigate the molecular basis for this discordance between mRNA and protein, we analyzed a public RNA‐seq time course dataset of monocyte‐to‐macrophage differentiation. Exon‐level quantification revealed that while most ADAR1 exons decreased or remained stable over the 7‐day differentiation period, a single exon corresponding to an alternative transcription start site (TSS), 20 kb upstream from the canonical TSS, exhibited a marked and selective increase (Figure S2H). Notably, transcripts initiated from this alternative TSS retain the capacity to encode both the p110 and p150 isoforms (Figure S2I), indicating that promoter choice modulates 5′‐UTR architecture rather than impacting isoform production.

Together, these data suggest that myeloid cells and particularly macrophages display high protein levels of ADAR1 in metabolic tissues and that this elevated protein expression can be recapitulated in vitro, likely driven by selective usage of an alternative TSS.

### 2.2 ADAR1 Binds and Edits Endocytic, Lysosomal, and Lipid Handling Genes in Macrophages

**FIGURE 2:**
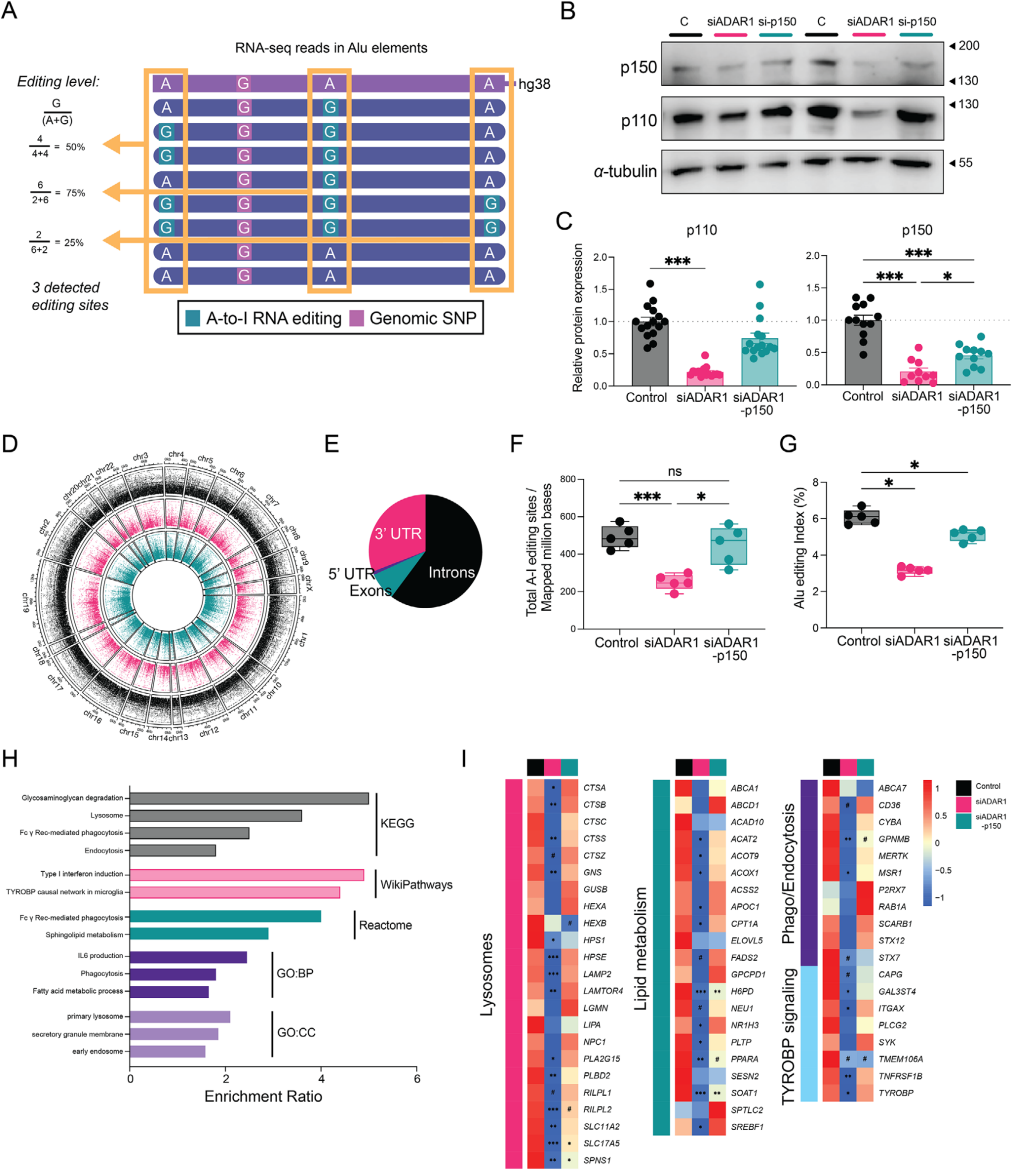
**ADAR1 binds and edits endocytic, lysosomal, and lipid handling genes in macrophages**. (**A**) Schematic illustrating detection and quantification of A‐to‐I RNA editing sites within Alu elements from RNA‐seq reads, highlighting editing event frequency. (**B, C**) Representative western blot analysis and quantification of ADAR1p110 and p150 relative protein expression in siADAR1, si‐ADAR1p150, and control‐transfected MDMs (*n* = 12–16). *α*‐tubulin was used as the loading control. (**D**) Circos plot depicting chromosomal distribution and frequency of detected A‐to‐I RNA editing events across experimental conditions. Each track represents one transfection condition. (**E**) Pie chart illustrating the distribution of A‐to‐I RNA editing sites within Alu elements across functional genomic regions. (**F, G**) Quantification of A‐to‐I RNA editing events in Alu elements, normalized to mapped sequencing reads, and corresponding Alu editing index in transfected MDMs (*n* = 5). (**H**) Functional pathway analysis of 1174 genes with extensive A‐to‐I RNA editing (>50 editing events) across indicated databases in transfected MDMs. (**I**) Scaled heatmap representing the normalized mean of A‐to‐I editing events within genes associated with the indicated functions in transfected MDMs (*n* = 5). Individual donor data are presented in Figure S3I. Box‐and‐whisker plots display min–max range, interquartile range (25th–75th percentiles), and median. Data in bar plots are presented as the mean ± SEM. Statistical significance was determined by one‐way ANOVA (**C**: p110, **F**) or mixed‐effects analysis (**C**: p150) or linear mixed‐effects models with donor as a random effect (**I**) followed by Tukey's multiple comparisons test, or one‐tailed Wilcoxon signed‐rank test (**G**). Enriched pathways with Benjamini–Hochberg (BH)‐adjusted *p*‐values < 0.05 are visualized (**H**). ^#^ *p* < 0.1, *p < 0.05, **p < 0.01, and ***p < 0.001.

The macrophage‐enriched expression of ADAR1 probed us to investigate the functional role of ADAR1 in the in vitro human MDM model. We performed RNA‐seq and applied a computational pipeline to detect A‐to‐I RNA editing events within Alu regions (Figure 2A). ADAR1 was silenced using siRNA targeting either both ADAR1 isoforms or the p150 variant

 specifically (Figure 2B,C; Figure S3A–D) without impacting cell viability or apoptosis (Figure S3E,F). Across all conditions, we identified 221,237 A‐to‐I RNA editing events in 8,609 genes in macrophages (Figure 2D), predominantly located within intronic and 3′‐UTR regions (Figure 2E), consistent with prior reports [10, 24]. Furthermore, 91% of detected sites overlapped with previously annotated editing sites in the REDI database [25], supporting the robustness of our pipeline. As expected, ADAR1 depletion resulted in a significant reduction in the number of detected A‐to‐I sites normalized to sequencing depth and in global Alu editing activity (Figure 2F,G). An illustrative example within the 3′‐UTR Alu region of CPT1A demonstrates a reduction in RNA editing sites and editing frequency upon ADAR1 knockdown (Figure S3G). Globally, the decrease of RNA editing rates and sites was modest upon p150‐specific knockdown, possibly due to the lower knockdown efficiency compared with the siRNA targeting both isoforms (Figure 2A,B; Figure S3A–D).

To comprehensively characterize the A‐to‐I RNA editome in macrophages, we focused on genes exhibiting extensive editing across donors. Notably, 1,174 genes exhibited more than 50 RNA editing events in our samples, prompting an overrepresentation analysis across multiple functional annotation databases (Figure S3H). Pathway enrichment analysis revealed significant overrepresentation of IL‐6 signaling and type I IFN responses (Figure 2H), aligning with previously characterized RNA editing targets [26, 27]. Additionally, functional enrichment analyses revealed enrichment in pathways related to endocytosis, phagocytosis, lysosomal function, lipid metabolism, and TYROBP signaling (Figure 2H). Consistent with the global editing reduction, ADAR1 silencing significantly reduced editing across numerous genes involved in macrophage scavenging and metabolic functions (Figure 2I; Figure S3I). Notably, siRNA targeting both isoforms exhibited a more pronounced effect compared with p150‐specific knockdown (Figure 2I; Figure S3I). Predicted ADAR1 substrates included lysosomal proteases (*CTSB*, *CTSS*, and *HPSE*), scavenger receptors (*CD36, MSR1*, and *MERTK*), TYROBP pathway components (*TYROBP* and *SYK*), and genes involved in lipid metabolism and regulation (*CPT1A*, *SOAT1*, *NR1H3*, *SREBF1*, and *PPARA*). Collectively, these data indicate that ADAR1 binds and edits core macrophage functional pathways related to scavenging and lipid handling.

### 2.3 ADAR1 Influences the Expression of Endocytic, Lysosomal, and Lipid Handling Genes in Macrophages

**FIGURE 3:**
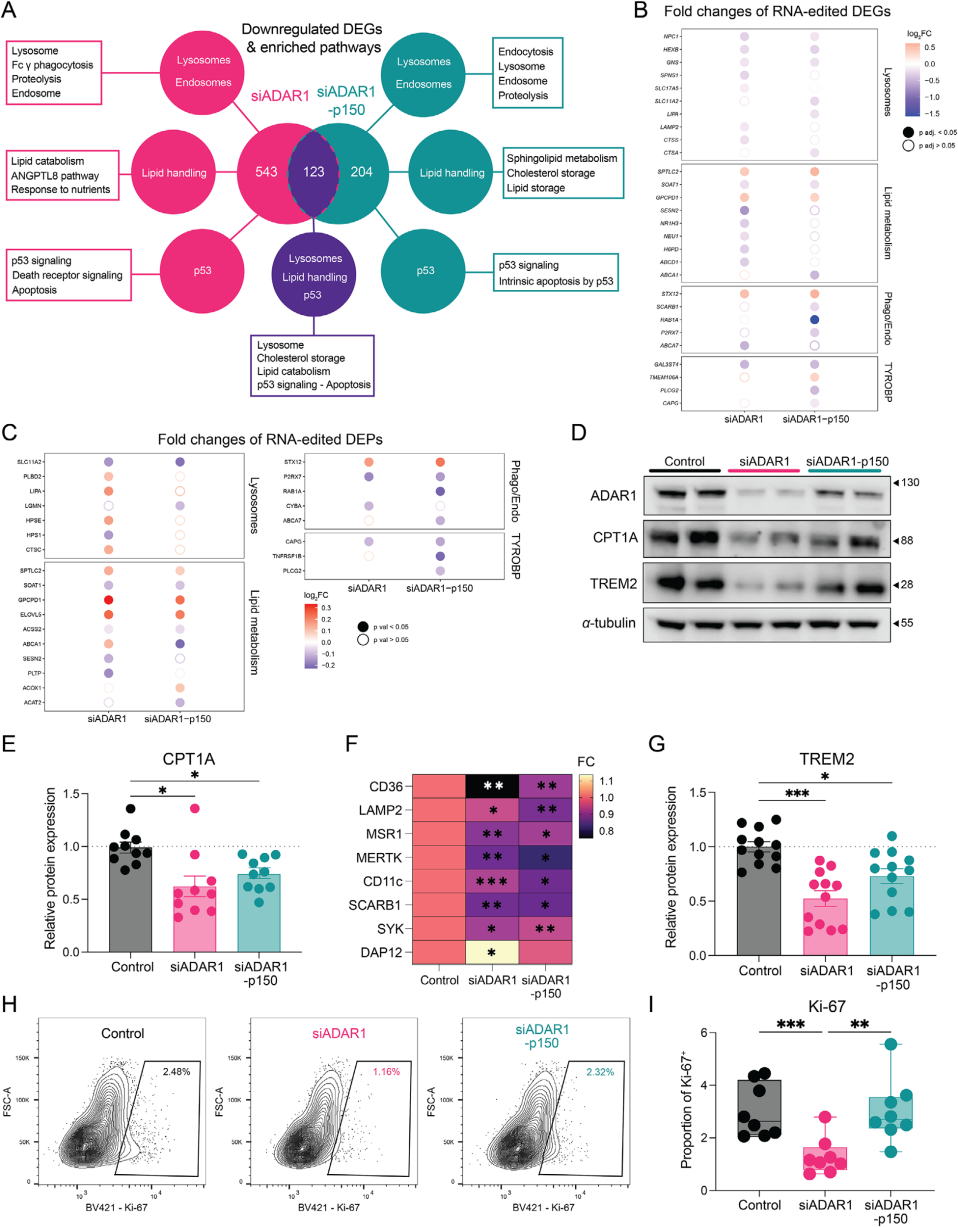
**ADAR1 influences the expression of endocytic, lysosomal, and lipid handling genes in macrophages**. (**A**) Venn diagram depicting the number of downregulated DEGs and their overlap in siADAR1 and siADAR1p150‐transfected MDMs compared with control‐transfected MDMs. Summarized pathways of overrepresentation analysis for each indicated comparison are visualized. (**B**, **C**) Dot plot displaying log2 fold changes of RNA editing DEGs (**B**) and DEPs (**C**) across transfected conditions, clustered by indicated functions. (**D**) Representative western blot of ADAR1, CPT1A, and TREM2 protein expression in 72 h posttransfected MDMs. (**E, G**) Bar plots showing fold changes in CPT1A (**E**; *n* = 10) and TREM2 (**G**; *n* = 12) protein levels from three independent experiments. *α*‐tubulin was used as the loading control. (**F**) Heatmap showing fold changes in protein expression of surface and intracellular markers (CD36, LAMP2, MSR1, MERTK, CD11c, SCARB1, DAP12, Syk) measured by flow cytometry (gMFI) in 72 h posttransfected MDMs from two independent experiments (*n* = 5–7). (**H**, **I**) Representative flow cytometry plots and quantification of Ki‐67^+^ MDMs from two independent experiments (*n* = 8). Data in bar plots are presented as mean ± SEM. Box‐and‐whisker plots display min–max range, interquartile range (25th–75th percentiles), and median. Statistical significance was determined by one‐way ANOVA with Tukey's post hoc test (**E**–**G**, **I**). Enriched pathways with Benjamini–Hochberg (BH)‐adjusted *p*‐values < 0.05 are visualized. (**A**) *p < 0.05, **p < 0.01, and ***p < 0.001.

To determine whether ADAR1 regulates the gene expression of the predicted target substrates, we performed differential expression analysis following ADAR1 depletion. Principal component analysis (PCA) demonstrated clear separation between control and ADAR1 knockdown conditions (Figure S4A). We identified 1,125 and 541 differentially expressed genes (DEGs) in ADAR1‐ and p150‐specific knockdown macrophages, respectively (Figure S4B–D). Of those, 249 DEGs (126 upregulated and 123 downregulated) overlapped between the two knockdown conditions, exhibiting a strong correlation in magnitude and directionality of expression changes (Figure S4E,F). Overrepresentation analysis of downregulated DEGs revealed significant enrichment in lysosomal, endosomal, phagocytic and lipid‐handling pathways for both siRNA conditions (Figure 3A). Consistently, shared downregulated genes were also enriched in lysosomal proteins, lipid, and cholesterol metabolism, suggesting a common molecular program downstream of ADAR1 depletion (Figure 3A). Interestingly, IFN signaling, a commonly affected pathway in ADAR1 knockdown cells [14, 28, 29], was not upregulated in our analysis.

Integration of editome and transcriptomic data identified 28 of 63 RNA editing substrates whose expression was altered upon ADAR1 depletion (Figure 3B). Notably, eight genes (*NPC1*, *HEXB*, *GNS, SPLTC2*, *SOAT1*, *GPCPD1*, *STX12*, and *GAL3ST4*) were shared between both silencing conditions (Figure 3B). These findings support a functional link between ADAR1 and core macrophage programs.

Given that A‐to‐I RNA editing can modulate protein expression post‐transcriptionally through effects on mRNA stability [30], nuclear retention [31], or alternative splicing [32], we next assessed whether ADAR1 depletion leads to detectable proteomic changes. We performed tandem mass tag (TMT)‐based quantitative proteomics at 48 h post‐transfection. PCA showed modest separation between control and ADAR1‐depleted macrophages, and only a limited number of proteins met stringent global significance thresholds (| Fold change | ≥10%, FDR < 0.1) (Figure S5A,B). These modest effects may reflect the early time point analyzed, at which protein turnover may lag transcriptional and post‐transcriptional changes. To capture early pathway‐level proteomic alterations, we applied a permissive filtering strategy (*p* < 0.05, | fold change | >7%), identifying 459 and 706 differentially expressed proteins (DEPs) in pan‐ADAR1 and p150‐specific knockdowns, respectively (Figure S5C,D). Pathway enrichment analysis revealed upregulation of lysosomal, endocytic, and cholesterol metabolism pathways in pan‐ADAR1 knockdown macrophages, whereas proteins downregulated upon p150‐specific silencing were preferentially associated with endocytosis and phagocytosis (Figure S5E,F). Notably, 17 DEPs involved in lipid handling, lysosomal function, phago/endocytosis, and TYROBP signaling were also RNA‐editing targets, providing orthogonal support consistent with the ADAR1‐regulated functional network (Figure 3C). Moreover, cell cycle, mitosis and chromosome segregation were among the most highly enriched pathways in downregulated DEPs only in pan‐ADAR1 MDMs.

To further assess protein‐level changes not fully captured by global proteomics at early timepoints, we performed targeted antibody‐based analyses at 72 h post‐transfection for selected predicted RNA‐editing targets. Immunoblotting revealed reduced CPT1A levels upon ADAR1 silencing (Figure 3D,E). Flow cytometric analysis demonstrated decreased surface expression of CD36, MSR1, CD11c, SCARB1, and MERTK, alongside reduced intracellular Syk and LAMP2 levels, whereas DAP12 (*TYROBP*) expression was increased (Figure 3F). Given the central role of TREM2‐DAP12‐Syk signaling in macrophage lipid metabolism and efferocytosis [33, 34, 35, 36], we assessed TREM2 expression and observed a significant reduction upon ADAR1 silencing (Figure 3D,G). Lastly, we validated the impact of pan‐ADAR1 on proliferation by the decreased proportions of Ki‐67^+^ MDMs only upon pan‐ADAR1 silencing (Figure 3H,I).

Together, these transcriptomic and proteomic analyses indicate that ADAR1 depletion disrupts the computationally predicted RNA‐editing network governing macrophage lipid handling, scavenging, lysosomal function, TYROBP‐associated pathways, and proliferation.

### 2.4 ADAR1 impacts Efferocytosis, Endocytosis, Lysosomal Function, and Lipid Metabolism

**FIGURE 4:**
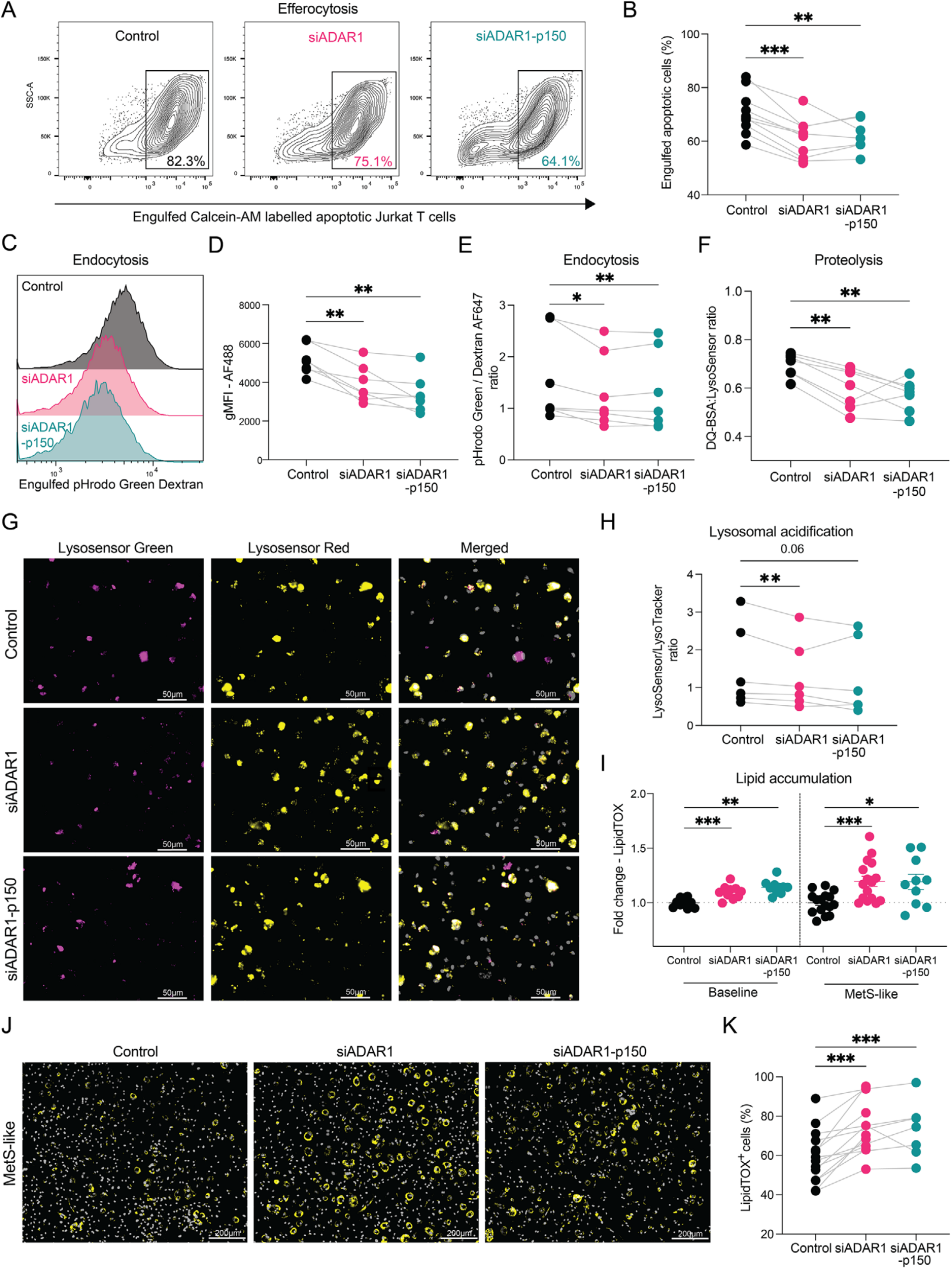
**ADAR1 impacts efferocytosis, endocytosis, lysosomal function, and lipid metabolism in macrophages**. (**A**, **B**) Representative flow cytometry plots and quantification of siADAR1‐, si‐ADAR1p150‐, and control‐transfected MDMs incubated with Calcein AM‐labelled apoptotic Jurkat cells for 60 min. Data are from three independent experiments (*n* = 7–10). (**C**, **D**) Representative histograms and quantification of engulfed pHrodo Green Dextran (10,000 MW) in transfected cells after 60 min incubation from two independent experiments (*n* = 7). (**E**) Paired dot plot of the pHrodo Dextran Green:Dextran AF647 ratio in transfected MDMs incubated for 60 min from two independent experiments (*n* = 7). Dextran AF647 particles were used to normalize for the total endocytotic uptake. (**F**) Paired dot plot of the DQ‐BSA AF647: LysoSensor Green ratio in transfected MDMs from two independent experiments (*n* = 7). MDMs were incubated with DQ‐BSA AF647 for 60 min, with LysoSensor Green added during the last 30 min to normalize for the functional lysosomal content. (**G, H**) Representative 40× live‐cell fluorescent images and quantification of transfected MDMs stained with LysoSensor Green (purple), LysoTracker Red (yellow), and Hoeschst 33342 (grey) for 30 min. Scale bar, 50 µm. Fluorescent intensity for each dye was quantified in five representative images per condition across all detected LysoTracker^+^ cells (*n* = 6). (**I**) Dot plots showing fold change in LipidTOX^+^ fluorescence in MDMs after 48 h transfection (*n* = 10) or additional overnight treatment with MetS‐like (*n* = 10–16) from four independent experiments. (**J**, **K**) Representative 20× fluorescent images and percentage quantification of LipidTOX^+^ in transfected MDMs after overnight MetS‐like treatment from three independent experiments (*n* = 7–10). Scale bar, 200 µm. Data are presented as the mean ± SEM or paired dot plots. Statistical significance was determined by one‐way ANOVA (**D**–**F**, **H**, **I**) or mixed‐effect analysis (**B**, **I, K**) followed by Tukey's multiple comparisons test.

To evaluate the functional relationship between ADAR1 and prototypical macrophage processes, we assessed efferocytosis, endocytosis, lysosomal activity, and lipid handling following ADAR1 depletion. To assess efferocytic capacity, Calcein AM–labelled apoptotic Jurkat T cells were incubated with either pan‐ADAR1 or p150 isoform‐specific knockdown macrophages for 1 hour. Both knockdowns resulted in a modest but significant ∼15% reduction in engulfment, indicating a contribution of ADAR1 to efferocytosis (Figure 4A,B). Phagocytic capacity was evaluated using pHrodo *E. coli* beads, which fluoresce upon phagosomal acidification. While total particle uptake was not significantly altered (Figure S6A,B), a slight reduction in fluorescence intensity was observed in ADAR1‐deficient macrophages (Figure S6C,D), suggesting impaired acidification. To further examine endosomal acidification, we used pHrodo  Green dextran, which revealed a more pronounced decrease in fluorescence intensity upon ADAR1 knockdown (Figure 4C,D). Co‐incubation with a pH‐insensitive dextran control confirmed that the reduced signal reflected impaired acidification rather than decreased endocytic uptake (Figure 4E). Lysosomal proteolytic function was assessed using DQ‐BSA in combination with LysoSensor to normalize for lysosomal content. ADAR1 depletion led to reduced proteolytic activity (Figure 4E), indicating compromised lysosomal degradation capacity independent of cargo size. Consistent with this, live‐cell immunofluorescence assays using LysoSensor (pH sensitive) and LysoTracker (pH insensitive) probes revealed a reduced lysosomal acidification ratio in both ADAR1 siRNA conditions. (Figure 4G,H). As a positive control, treatment with chloroquine, a lysosomotropic agent, recapitulated the expected defects in endocytosis, lysosomal acidification and proteolytic activity (Figure S6E).

Finally, to assess lipid handling, ADAR1‐deficient macrophages were exposed overnight to a metabolic syndrome‐like (MetS‐like) medium containing free fatty acids (FFA), high levels of insulin, fructose, and glucose. Flow cytometric analysis using LipidTOX staining revealed increased lipid accumulation in ADAR1 knockdown macrophages compared with controls under MetS‐like conditions (Figure 4I). Notably, elevated lipid accumulation was also observed in ADAR1‐silenced macrophages under basal conditions 48 h post‐transfection (Figure 4I). These findings were corroborated by immunofluorescence analysis, which showed a higher proportion of LipidTOX^+^ cells in ADAR1 knockdown macrophages following MetS‐like treatment (Figure 4J,K). In summary, these functional assays provide mechanistic evidence that ADAR1 supports efferocytosis, endocytosis, lysosomal acidification, and lipid metabolism.

### 2.5 p150 Overexpression Rescues Lysosomal, Scavenging and Lipid Metabolism Pathways in p110‐Deficient Macrophages

**FIGURE 5:**
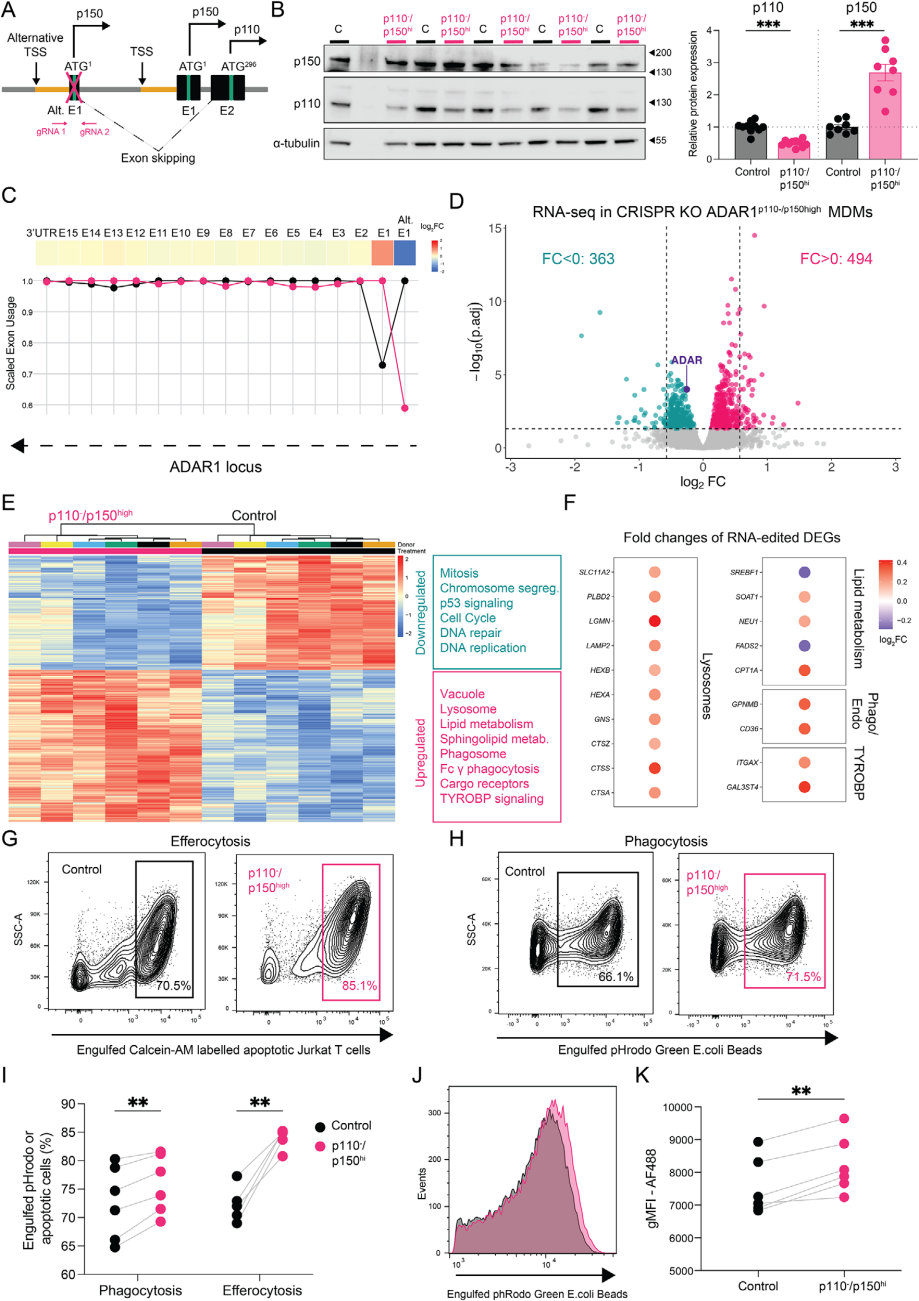
**p150 overexpression rescues lysosomal, scavenging, and lipid metabolism pathways in p110‐deficient macrophages**. (**A**) Schematic illustrating the canonical and alternative TSS of ADAR1 and CRISPR guide RNAs‐targeting strategy. (**B**) Representative western blot analysis and quantification of ADAR1p110 and p150 relative protein expression in ADAR1^p110−^/p150high, and CRISPR control (non‐targeting) MDMs from two independent experiments (*n* = 8–10). *α*‐tubulin was used as the loading control. (**C**) (**Top**) Heatmap showing log2 fold changes (p110^−^/p150^high^/Control) for each differentially expressed ADAR1 bins (adj. *p*‐value < 0.05). (**Bottom**) Scaled exon usage plots for control (black) and p110^−^/p150^high^ (pink). DEXSeq bin‐level results were aggregated to exon‐level averages weighted by bin length. (**D**) Volcano plot depicting DEGs in ADAR1p110^−^/p150^high^ MDMs relative to controls (*n* = 6). The number of DEGs (adj. *p*‐value < 0.05) is indicated for each direction. (**E**) Heatmap showing normalized counts of DEGs in ADAR1p110^−^/p150^high^ relative to control and overrepresented pathways across multiple functional databases, shown separately for downregulated and upregulated. Values are shown after donor batch correction. (**F**) Dot plot displaying log2 fold changes of RNA editing DEGs, clustered by indicated functions. (**G**–**I**) Representative flow cytometry plots and quantification of indicated MDMs engulfing Calcein AM‐labelled apoptotic Jurkat cells (**G**; *n* = 5) or engulfing pHrodo Green‐labeled *E. coli (* **H**; *n* = 6) after 60 min incubation. (**J**, **K**) Representative histograms and quantification of engulfed pHrodo Green‐labeled *E. coli* after 60 min incubation. Data are presented as the means ± SEM or paired dot plots. Statistical significance was determined by a ratio paired t‐test (**B**, **I**, **K**) or negative binomial Wald test. Enriched pathways with Benjamini–Hochberg (BH)‐adjusted *p*‐values < 0.05 are visualized (**E**) *p < 0.05, **p < 0.01, and ***p < 0.001.

To investigate the role of the alternative TSS of ADAR1 and its impact on macrophage differentiation, we attempted CRISPR‐mediated disruption of the alternative TSS in monocytes and differentiated them into MDM (Figure 5A). Surprisingly, with this approach, p110 protein levels were reduced, while p150 levels were compensatory increased (Figure 5B). Exon usage analysis revealed that this compensation was driven by enhanced expression from the canonical TSS rather than an isoform switch (Figure 5C). Thus, this setup effectively represents an isoform‐specific rescue model, with p150 overexpressed and p110 reduced (p110^−^/p150^high^). To dissect the functional contributions of each isoform, we performed RNA‐seq to determine whether the transcriptional networks affected by pan‐ADAR1 or p150‐specific silencing could be reversed or attributed to individual isoforms.

Differential expression analysis identified 494 upregulated and 363 downregulated DEGs in the p110^−^/p150^high^ MDMs (Figure 5D). Upregulated genes were enriched for pathways associated with lysosomal function, lipid metabolism, phagocytosis, and TYROBP signaling, whereas downregulated genes were enriched for mitosis, DNA replication, and cell cycle regulation (Figure 5E). Notably, 19 predicted RNA‐editing targets involved in scavenging and lipid metabolism were differentially expressed, with the majority showing upregulation (Figure 5F). Several targets downregulated upon ADAR1 silencing, including HEXB, GNS, CTSS, NEU1, and GAL3ST4 at the mRNA level, CD36, CPT1A, and CD11c at the protein level, and SOAT1, SLC11A2, and LAMP2 at both mRNA and protein levels, were restored in the p110^−^/p150^high^ condition. Functionally, p110^−^/p150^high^ MDMs exhibited enhanced efferocytic and phagocytic capacity, as measured by uptake of apoptotic cells and pHrodo‐labelled beads (Figure 5G–I). Phagosomal acidification was also elevated, indicating that both the uptake and digestion of cellular material are increased in this condition (Figure 5J,K).

Together, these findings, integrated with RNA‐seq, proteomics, and functional data from pan‐ADAR1 and p150‐specific silencing, indicate isoform‐specific roles: p150 drives lysosomal function, scavenging, and lipid metabolism, whereas p110 primarily regulates mitotic and cell cycle pathways.

### 2.6 ADAR1 is Enriched in Macrophage Subpopulations With Specialized Functional Programs

**FIGURE 6:**
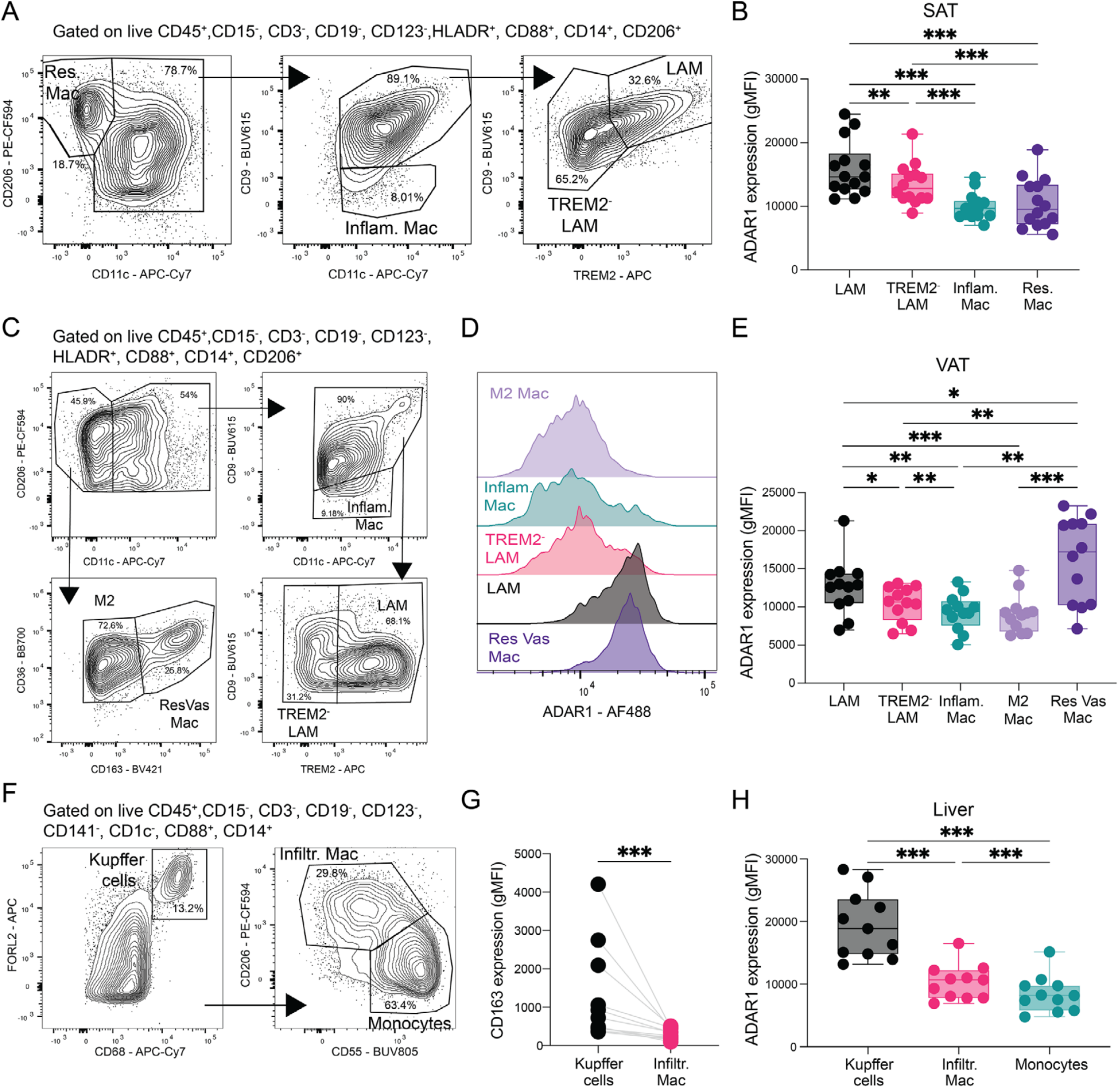
**ADAR1 is enriched in macrophage subpopulations with distinct functional macrophage programs across tissues**. (**A, B**) Representative flow cytometry gating strategy and summary data for ADAR1 expression (gMFI) in indicated SVF cell populations from SAT biopsies (*n* = 14). (**C–E**) Representative flow cytometry gating strategy, histograms, and summary data for ADAR1 expression (gMFI) in indicated SVF cell populations from VAT biopsies (*n* = 12). (**F, G, H**) Representative flow cytometry gating strategy and summary data for CD163 and ADAR1 expression (gMFI) in indicated NPCs from perfused human livers (*n* = 11). Box‐and‐whisker plots display the range (min–max), interquartile range (25th–75th percentiles), and median. Statistical significance was determined by one‐way ANOVA followed by Tukey's multiple comparisons test (**B**, **E**, **H**) or Wilcoxon signed rank test (**G**).

We next investigated whether ADAR1 expression is associated with specific macrophage subpopulations characterized by the ADAR1‐regulated spectrum. To this end, we delineate adipose tissue macrophage (ATMs) subsets from our flow cytometry data from SAT and VAT samples of patients with obesity (Figure 1H,I). Our gating strategy was cross‐referenced with published sc‐ and snRNA‐seq atlases (Table S1) [37, 38, 39, 40]. Within Infiltr. Mac (CD11c^+^/CD206^+^), we stratified cells based on CD9 and TREM2 expression, identifying three populations: CD9^−^, CD9^+^/TREM2^−^, and CD9^+^/TREM2^+^ (Figure 6A). Across both adipose depots, the CD9^+^/TREM2^+^ subset exhibited significantly higher CD36 expression, supporting its classification as Lipid‐associated macrophages (LAMs) (Figure S7A,B). LAMs are known to exhibit enhanced efferocytic and proliferative capacity, lipid handling, and enriched lysosomal features [41, 42, 43]. The CD9^+^/TREM2^−^ (TREM2^−^ LAM) subset likely represents a transitional macrophage CD9^+^ population, previously identified in sc‐analyses of VAT from patients with obesity and metabolic dysfunction‐associated steatotic liver disease (MASLD) [38]. In addition, we also identified a macrophage population (CD11c^+^/CD9^−^), classified as inflammatory macrophages (Inflam. Mac). This subset was marked by high S100A9 expression and intermediate CD206 levels (Figure S7C–E), consistent with previously published single‐cell datasets [38, 39]. Although the data suggest a continuum marked by decreasing S100A9 and increasing CD206/HLA‐DR expression from monocytes to Inflam. Mac to CD9 Mac (Figure S7F), we cannot exclude the possibility that Inflam. Mac may represent a transitional or recently differentiated macrophage state. Among all ATM subpopulations, LAMs displayed the highest ADAR1 expression in SAT (Figure 6B) and VAT (Figure S7G). Notably, Res. Mac (CD206^+^/CD11c^−^) from VAT also displayed elevated ADAR1 expression, prompting further stratification. Consistent with prior studies identifying CD163^high^ CD206^+^ macrophages enriched in VAT [37] and associated with lysosomal and lipid metabolic programs [38], we subdivided Res. Mac into resident vasculature‐associated macrophages (Res Vac Mac) and CD163^low^ M2 macrophages (Figure 6C). ADAR1 expression was highest in Res Vac Mac, exceeding levels observed in LAMs, whereas M2 macrophages exhibited significantly lower ADAR1 expression (Figure 6D,E). Collectively, these data suggest that ADAR1 is selectively enriched in ATM subsets specialized in lipid metabolism, scavenging properties, and proliferative potential.

To determine whether this association extends beyond adipose tissue, we examined macrophage subpopulations in the human liver. Kupffer cells (KCs), the resident liver macrophages, exhibit robust efferocytotic and proliferative capacity [44, 45, 46], active lipid metabolism [47, 48, 49], and pronounced lysosomal activity [50, 51]. KCs were identified using FOLR2, a marker previously shown to selectively label this resident population (Figure 6F; Figure S7H) [52]. The FOLR2^high^ population expressed high levels of CD68 and CD163, confirming its resident identity (Figure 6F,G). ADAR1 levels were significantly elevated in KCs compared with Infiltr. Mac (Figure 6H). Consistent with our previous analysis, both KCs and Infiltr. Mac exhibited significantly higher ADAR1 levels than monocytes (Figure 6H). Together, these findings demonstrate that ADAR1 expression correlates with macrophage subpopulations characterized by scavenging, lipid metabolic, lysosomal, and proliferative activity, and this association is conserved across metabolic tissues.

### 2.7 ADAR1 is Dynamically Regulated in MASLD and Macrophage‐specific ADAR1 Ablation Exacerbates Lipid Accumulation

**FIGURE 7:**
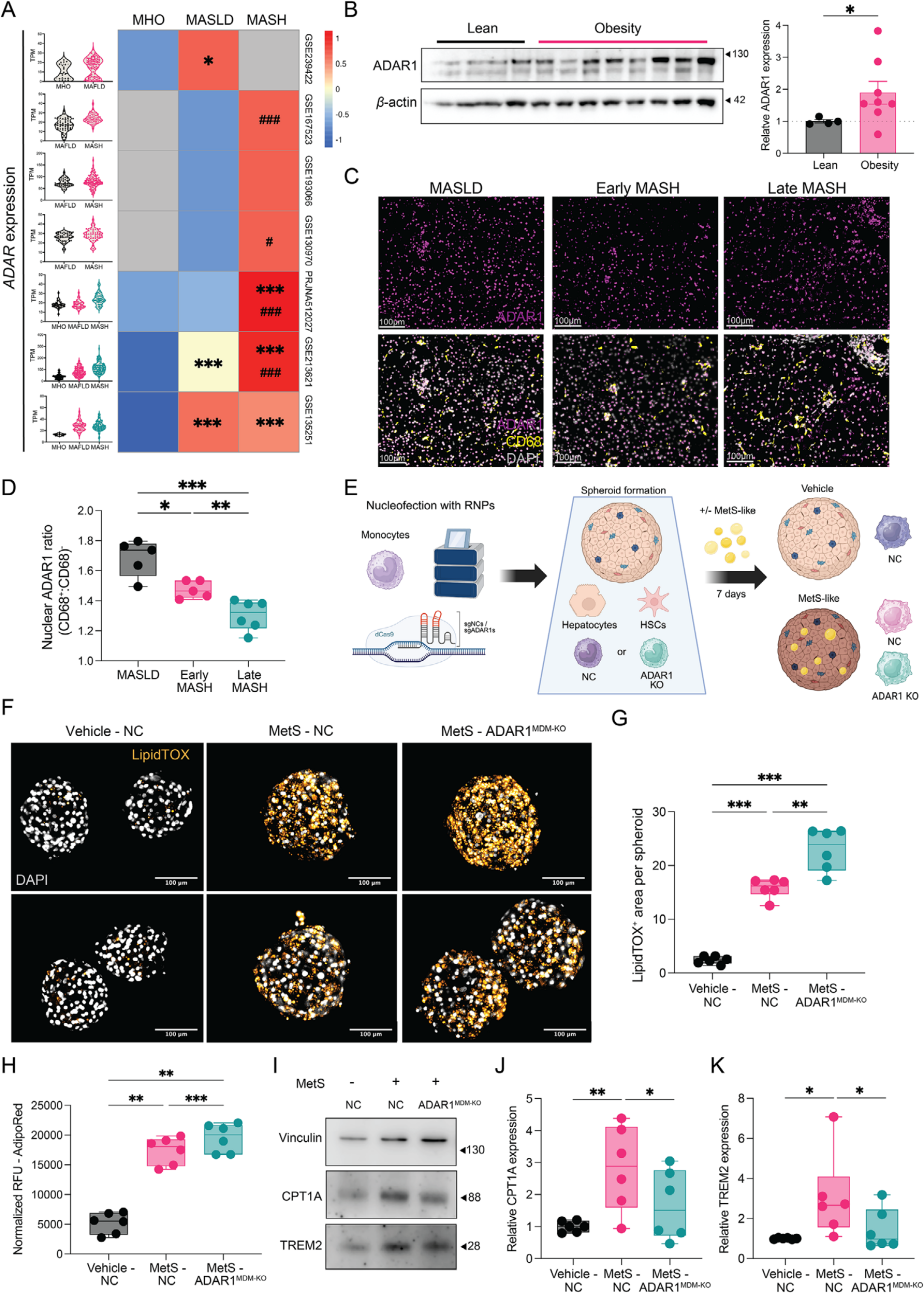
**ADAR1 is dynamically regulated in MASLD, and macrophage‐specific ADAR1 ablation exacerbates lipid accumulation**. (**A**) Violin plots and a heatmap depicting *ADAR* mRNA levels (TPM) in human liver tissue from MHO, MASLD, and MASH across multiple RNA‐seq datasets. The heatmap displays median values per group; nonapplicable groups are shown in grey. *Indicates comparison to MHO and ^#^indicates comparison to MASLD. (**B**) Western blot analysis and quantification of ADAR1 expression in liver biopsies from normal‐weight individuals (*n* = 4) and patients with obesity (*n* = 8). *β*‐actin was used as the loading control. (**C**, **D**) Representative 20× immunofluorescence images of FFPE liver from MASLD (*n* = 5), early MASH (*n* = 5), and late MASH patients (*n* = 6), stained for ADAR1 (purple), CD68 (yellow), with DAPI nuclear counterstain (grey). Scale bar, 100 µm. Box‐and‐whisker plots show normalized nuclear ADAR1 fluorescence intensity (FI) as the CD68^+^/CD68^−^ ratio. Fluorescent intensity was normalized to nucleus size. (**E**) Schematic overview of the 3D organotypic hepatic spheroid model composed of PHH, HSC, and CRISPR‐edited monocytes (sg‐negative controls or sg‐ADAR1s). Following spheroid formation, cultures were exposed to MetS‐like media or vehicle for 7 days. (**F**) Representative 20× whole‐mount immunofluorescence images of hepatic spheroids stained with LipidTOX Deep Red (orange) and Hoeschst 33342 (grey) from two representative donors in the indicated treatments. (**G**) Box‐and‐whisker plots showing LipidTOX^+^ area in indicated treatment conditions from two independent experiments (*n* = 6). Each biological replicate represents the mean of individual spheroids (*n* = 7–10). (**H**) Box‐and‐whisker plots showing normalized RFU values of AdipoRed in dissociated hepatic spheroids from two independent experiments (*n* = 6). Each biological replicate represents the mean of individual spheroids (*n* = 3–12). (**I–K**) Representative western blot analysis and quantification of CPT1A and TREM2 relative protein expression in hepatic spheroids under indicated treatment conditions from two independent experiments (*n* = 6). Vinculin was used as the loading control. Box‐and‐whisker plots display min–max range, interquartile range (25th–75th percentiles), and median. Statistical significance was determined by the Mann–Whitney test (**A**) or unpaired t‐test with Welch correction (**B**, **D**) or one‐way ANOVA followed by Tukey's multiple comparisons test (**G**, **H**) or ratio p‐test (**J**, **K**). *p < 0.05, **p < 0.01, and ***p < 0.001.

Macrophage lipid metabolism and scavenging functions play central roles in the progression of MASLD [41, 53], prompting us to examine how ADAR1 expression and activity are regulated in clinical tissue samples. In liver biopsies, *ADAR1* mRNA expression progressively increased from metabolically healthy patients with obesity (MHO) to MASLD and further in metabolic dysfunction‐associated steatohepatitis (MASH) (Figure 7A). Consistently, protein analysis confirmed elevated ADAR1 levels in obese compared with lean livers (Figure 7B), with a positive correlation to HOMA‐IR, suggesting early induction during metabolic dysfunction (Figure S8A). Global A‐to‐I RNA editing followed a similar trend, rising from MHO to MASLD but declining in MASH (Figure S8B,C). As liver macrophages accumulate across the MASLD spectrum [54, 55, 56], we next assess their contribution to the ADAR1 upregulation. Immunofluorescence analysis revealed reduced ADAR1 expression in CD68^+^ cells relative to CD68^−^ cells in MASH (Figure 7C,D), indicating that nonmacrophage cells may be the principal contributors to ADAR1 upregulation in advanced disease stages.

To validate a potential macrophage‐specific role of ADAR1 in MASLD, we employed a 3D organotypic primary human liver spheroid model comprised of primary human hepatocytes (PHH), hepatic stellate cells (HSC), and monocytes (Figure S8D). Seven days postseeding, HSCs and monocytes were integrated within the spheroids, as evidenced by expression of HSC marker α‐SMA and the macrophage markers CD163 and TREM2 (Figure S8E). Because monocytes typically undergo apoptosis in culture in the absence of differentiating factors [57, 58], sustained expression of CD163 and TREM2 indicates survival and differentiation into macrophages within the spheroid microenvironment, recapitulating features of monocyte infiltration and differentiation in vivo. Exposure of spheroids to the MetS‐like cocktail for 7 days resulted in a significant induction of both ADAR1 protein isoforms, mirroring the upregulation observed in clinical MASLD cohorts (Figure S8F). These data support the physiological relevance of this organotypic human tissue model for interrogating macrophage‐specific ADAR1 function in metabolic disease.

To assess the functional consequences of macrophage‐specific loss, we generated ADAR1‐deficient monocytes using a CRISPR‐based approach and confirmed reduced protein levels of total ADAR1 and p150 isoform in differentiated MDMs under M‐CSF conditions (Figure S8G–J). ADAR1 KO monocytes were incorporated into liver spheroids and exposed to MetS‐like cocktail (Figure 7E). Spheroids containing ADAR1‐deficient MDMs (ADAR1^MDM‐KO^) exhibited significantly exacerbated lipid accumulation compared with MetS control, as assessed by increased LipidTOX^+^ area and biochemical quantification (Figure 7F–H). Consistent with impaired lipid handling, spheroids harboring ADAR1‐deficient macrophages displayed reduced protein levels of CPT1A and TREM2 following MetS treatment (Figure 7I–K), suggesting compromised lipid oxidation and, potentially, efferocytic capacity.

### 2.8 ADAR1 is Dynamically Regulated in Response to Weight Gain and Loss in a Cell‐Specific Manner

**FIGURE 8:**
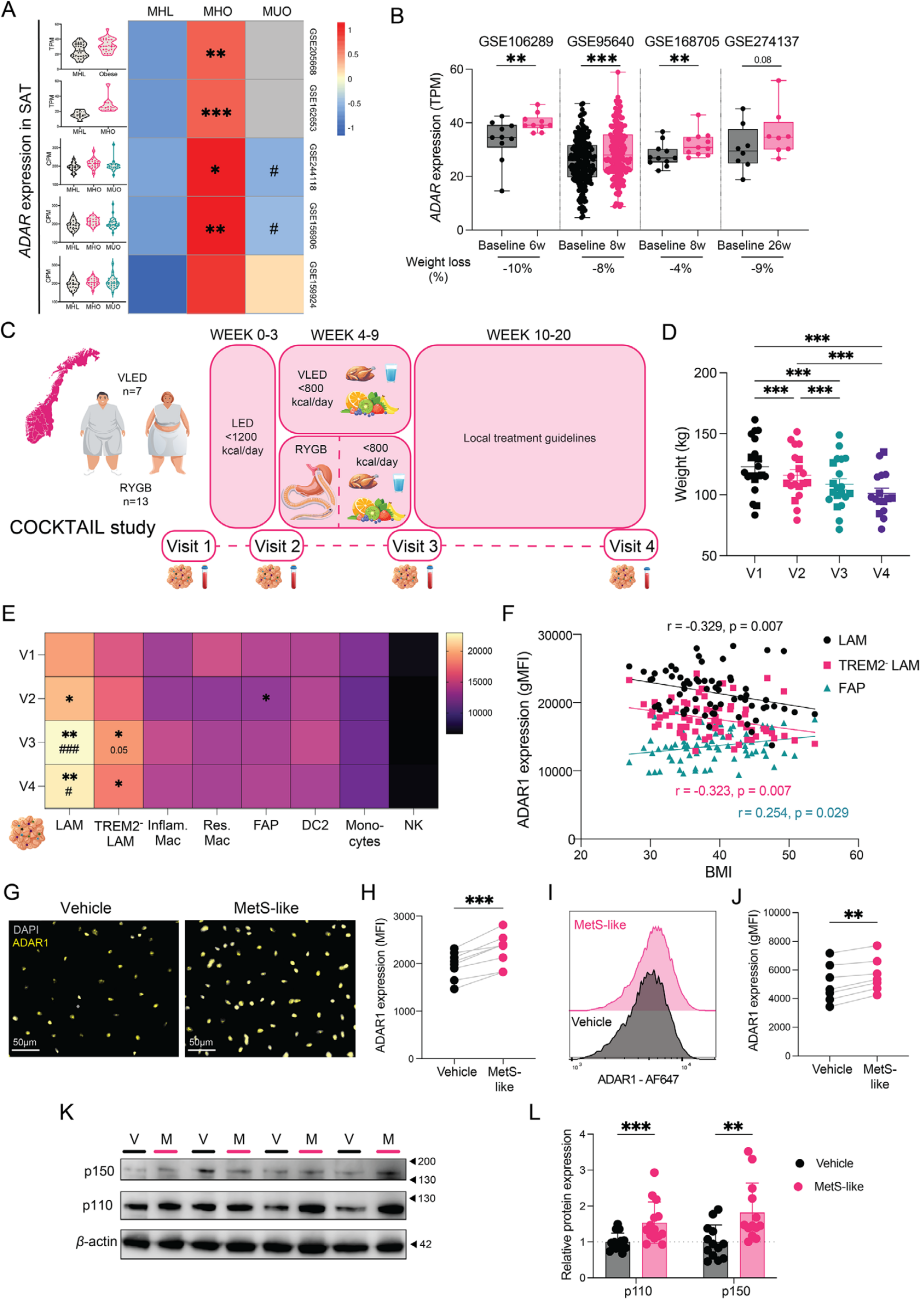
**ADAR1 is dynamically regulated in adipose tissue in response to weight gain and loss in a cell‐specific manner**. (**A**) Violin plots and a heatmap illustrating *ADAR1* mRNA levels (TPM or CPM) in SAT from MHL, MHO, and MUO individuals across five RNA‐seq datasets. Heatmap depicts median values per group; nonapplicable groups are displayed in grey. *Indicates the comparison to MHL and ^#^ the comparison to MHO. (**B**) Box‐and‐whisker plots of *ADAR1* mRNA levels (TPM) in SAT from patients with obesity achieving 4%–10% weight loss through caloric diet across four public RNA‐seq datasets. (**C)** Schematic overview of the COCKTAIL study, outlining study design, interventions, and timepoints. (**D**) Dot plots showing weight (kg) in patients undergoing VLED (*n* = 7; squares) and RYGB (*n* = 13; circles) across all visits. (**E**) Heatmap of median ADAR1 expression (gMFI) in indicated SVF cells from SAT biopsies at V1‐V4 timepoints in VLED (*n* = 7) and RYGB (*n* = 13) arms. (**F**) Correlation plot of ADAR1 expression (gMFI) and BMI in indicated cell populations. Spearman correlation coefficient (*r*) and *p*‐values are displayed in the graph. (**G, H**) Representative 40× fluorescent images and quantification of nuclear ADAR1 expression (MFI) of MDMs treated with MetS‐like and vehicle overnight. Scale bar, 50 µm. Fluorescent intensity per nucleus for ADAR1 was quantified in two representative images per condition at 20× magnification from three independent experiments (*n* = 8). (**I**, **J**) Representative flow cytometry histograms and quantification of ADAR1 expression (gMFI) in MDMs treated with MetS‐like or vehicle overnight from two independent experiments (*n* = 7). (**K, L**) Western blot analysis and quantification of ADAR1p110 and p150 protein expression in MDMs treated with MetS‐like or vehicle overnight, and summary data from five independent experiments (*n* = 13–15). *α*‐tubulin was used as the loading control. Data in bar and dot plots are presented as the means ± SEM. Box‐and‐whisker plots display the min–max range, interquartile range (25th–75th percentiles), and median. Statistical significance was determined by Mann–Whitney (**A**), Wilcoxon signed‐rank (**B**), one‐way ANOVA (**D**), mixed‐effect analysis (**E**), followed by Tukey's multiple comparisons test or ratio *p*‐test (**H**, **J**, **L**). *p < 0.05, **p < 0.01, and ***p < 0.001.

We next examined ADAR regulation in adipose tissue across metabolic states. Analysis of multiple SAT bulk RNA‐seq datasets from individuals with normal weight or obesity revealed that *ADAR1* expression was higher in MHO compared with metabolically healthy lean (MHL) individuals but declined in metabolically unhealthy patients with obesity (MUO) (Figure 8A). This indicates a nonlinear relationship between *ADAR1* levels and adipose metabolic health. Moreover, global RNA editing was reduced in MUO compared with MHL (Figure S9A). To assess whether *ADAR1* expression is dynamically modulated by weight loss, we examined SAT biopsies from four diet‐induced weight loss studies (4%–10% weight loss). Across all studies, both *ADAR1* expression and global RNA editing increased following intervention (Figure 8B; Figure S9B,C), indicating that ADAR1 levels and activity in adipose tissue are responsive to metabolic adaptation. To resolve cell‐type‐specific ADAR1 regulation during weight loss, we leveraged SAT SVF samples from a subset of participants with severe obesity in the COCKTAIL study (Figure 8C) [59]. The study protocol included a baseline assessment (Visit 1; V1), an initial 3‐week calorie restriction phase (Visit 2; V2), an intensified 6‐week caloric restriction period with or without Roux‐en‐Y gastric bypass (RYGB) (Visit 3; V3), and a follow‐up assessment after an additional 10 weeks (Visit 4; V4) (Figure 8C). To focus on weight loss‐associated changes, data from both intervention arms were pooled (Figure 8D). Strikingly, ADAR1 protein levels were selectively and robustly upregulated in LAMs across all timepoints, as well as in the TREM2^−^ LAM subset at V3 and V4 (Figure 8E). In contrast, ADAR1 expression remained unchanged in other SVF populations apart from a transient decrease observed in fibro‐adipogenic progenitors (FAPs) at V2. Consistent with this specificity, ADAR1 expression in LAM subsets inversely correlated with body mass index (BMI), whereas FAPs showed the opposite trend (Figure 8F). To determine whether LAM‐specific ADAR1 induction reflected recruitment of circulating monocytes or local microenvironmental regulation, we measured ADAR1 levels in matched peripheral immune cells. In contrast to adipose tissue, peripheral ADAR1 levels decreased after intervention and at follow‐up (Figure S9D), and positively correlated with BMI (Figure S9E), suggesting that local adipose cues, rather than monocyte influx, drive LAM‐specific ADAR1 elevation.

We next characterized shifts in ATM composition during weight loss (Figure S9F). LAMs expanded post‐intervention (V3) but declined by follow‐up (V4), whereas Res. Macs became the dominant ATM population at V4. Inflam. Mac proportions were not significantly altered by weight loss but positively correlated with BMI (Figure S9G). These dynamics mirror prior observations describing transient LAM accumulation and crown‐like structure (CLS) formation during early weight loss [60, 61, 62, 63], followed by restoration of Res. Mac populations during longer‐term tissue remodeling [63, 64]. Supporting this temporal shift, reanalysis of published datasets showed elevated *TREM2* expression in short‐term interventions (6–8 weeks; Figure S9H), and increased *CD163* expression following longer‐term loss (26 weeks; Figure S9I). Finally, enhanced adipocyte lipolysis during early weight loss has been proposed as a key driver of LAM expansion [62, 65]. To directly test whether elevated lipid availability induces ADAR1, we exposed MDMs to MetS‐like media in vitro. Acute MetS exposure robustly increased ADAR1 protein levels, as assessed by immunocytochemistry, western blot, and flow cytometry (Figure 8G–L).

Collectively, these findings indicate that ADAR1 is selectively upregulated in LAMs during early weight loss, potentially reflecting macrophage adaptation to a lipolytic adipose microenvironment.

## 3 Discussion

In this study, we define ADAR1 as a macrophage‐enriched regulator of core macrophage programs under both homeostatic and metabolic disease conditions. Beyond its established role in suppressing dsRNA‐mediated autoimmunity, our findings reveal that ADAR1 supports macrophage functions central to lipid handling, scavenging, and proliferation in an isoform‐specific manner, and that its expression and activity are dynamically modulated by the human metabolic state.

We demonstrate that human macrophages exhibit markedly higher ADAR1 protein levels than other immune and parenchymal cell types across tissues, with ADAR1 induction occurring during monocyte‐to‐macrophage differentiation. Notably, this enrichment is largely uncoupled from ADAR1 mRNA expression and is driven instead by selective usage of an alternative TSS during differentiation, providing a likely explanation for why macrophage‐specific ADAR1 enrichment has been underappreciated in single‐cell transcriptomic studies. While elevated ADAR1 protein levels during differentiation have been previously observed in THP‐1 and U937 models [66], the functional relevance has remained largely unexplored.

Interrogation of the A‐to‐I RNA editome in human MDMs revealed that ADAR1 binds and edits targets involved in efferocytosis, endocytosis, lysosomal activity, and lipid metabolism. Loss‐of‐function analyses supported by convergent transcriptomic, proteomic, and functional assays demonstrate that disruption of ADAR1 compromises these pathways. Importantly, these phenotypes occurred in the absence of aberrant IFN activation, indicating that ADAR1 contributes to macrophage functional programs independently of its canonical role in suppressing dsRNA‐driven autoimmunity [14, 16, 17]. Transcriptomic and functional analyses of isoform‐specific rescued macrophages indicated that p150 preferentially regulates lysosomal function, lipid metabolism, and phagocytic programs, whereas p110 predominantly influences cell cycle and proliferation. Notably, cell cycle and mitotic genes were not identified as ADAR1 RNA editing substrates, suggesting that p110 may regulate these processes through RNA‐editing‐independent mechanisms, consistent with previous observations [67, 68]. These mechanistic insights complement our observations that ADAR1 is enriched in macrophage subsets specialized for scavenging, lipid buffering, and with high proliferative potential across metabolic tissues, including LAMs in adipose tissue [36, 41, 42, 43], Res Vac Mac in VAT [38], and Kupffer cells in liver [44, 45, 46, 47, 48, 49, 50, 51]. Beyond macrophages, ADAR1 has also been implicated in lipid accumulation and metabolic regulation in cancer cell lines [69] and adipocytes [70], suggesting that its role in lipid handling may extend across cell types.

Given the central role of macrophage lipid metabolism, scavenging, and proliferation in metabolic disease [41, 42, 43], we next examined ADAR1 regulation in clinical samples. ADAR1 expression increased progressively with MASLD severity; however, macrophage‐specific ADAR1 levels declined relative to non‐macrophage cells in advanced MASH. This divergence suggests that while global ADAR1 expression may rise in response to metabolic stress, macrophages may lose this adaptive program during disease progression. Supporting this interpretation, macrophage‐intrinsic loss of ADAR1 in an organotypic 3D human liver spheroid model exacerbated lipid accumulation under metabolic stress. Reduced expression of CPT1A and TREM2, targets involved in lipid oxidation [71] and efferocytosis [53], was observed in both 3D liver spheroids and 2D MDM model, reinforcing the relevance of these pathways.

In adipose tissue, we identify a dynamic relationship between ADAR1 expression and metabolic state. Elevated ADAR1 levels in MHO may enhance macrophage lipid‐buffering capacity, whereas chronic inflammation and metabolic dysfunction appear to overwhelm this adaptive response. Consistent with this notion, weight loss interventions, known to restore metabolic homeostasis in adipose tissue and reshape the macrophage landscape [62, 64, 65, 72, 73], were associated with increased ADAR1 levels and activity. Notably, in our longitudinal COCKTAIL cohort, short‐term weight loss induced a robust, LAM‐specific upregulation of ADAR1 that inversely correlated with BMI. This response was not mirrored in circulating monocytes, indicating that ADAR1 regulation is driven by the local adipose microenvironment remodeling rather than monocyte recruitment. Although long‐term caloric restriction reduces total ATMs [63, 73], multiple human and murine studies report a paradoxical increase in LAMs and CLS during acute weight loss or following rapid weight fluctuations [60, 61, 62, 65]. This biphasic behavior was also evident in our cohort, with transient LAM expansion during acute weight loss followed by a decline at later time points. Enhanced lipolysis during acute weight loss has been proposed as a key driver of this response [62], and our in vitro data showing induction of ADAR1 expression following exposure of macrophages to FFAs support a mechanistic link between lipid availability and ADAR1 regulation. We speculate that LAM‐specific ADAR1 induction may contribute to efferocytosis of apoptotic adipocytes, a hallmark of early weight loss [65], consistent with the modest effects we observed on efferocytosis in 2D macrophages and the TREM2 reduction in 3D liver spheroids.

Collectively, our data support a model in which macrophage‐specific induction of ADAR1 enhances macrophage capacity to buffer lipotoxicity during periods of metabolic flux through coordinated regulation of lipid metabolism and scavenging pathways. This adaptive program may contribute to tissue homeostasis during weight loss and other dynamic metabolic states.

Our study has limitations. We do not distinguish RNA editing‐dependent from editing‐independent ADAR1 functions, and future studies using catalytically inactive mutants will be required to resolve these mechanisms. While our human data reveal strong associations, causal inference in vivo remains limited. Macrophage‐specific ADAR1 knockout mouse models could provide causal insights, particularly under high‐fat diet conditions. However, interspecies differences in ADAR1 biology, including the primate‐specific abundance and genomic distribution of Alu elements and higher RNA editing frequencies, necessitate cautious interpretation [10, 74]. Indeed, even conserved ADAR1‐dsRNA‐MDA5 pathways rely on divergent endogenous dsRNA repertoires between mice and humans [75], underscoring the importance of complementary human‐based systems.

In summary, we identify ADAR1 as a macrophage‐enriched post‐transcriptional regulator that supports lipid handling, scavenging, and proliferative capacities beyond its canonical IFN‐suppressive role, in an isoform‐specific manner. These findings highlight post‐transcriptional regulation as a central axis of macrophage plasticity and provide a foundation for future studies dissecting how these regulatory layers shape immune function in homeostasis and metabolic disease.

## 4 Material and Methods

### 4.1 Human Cohorts

Human liver samples for flow cytometry analysis of non‐parenchymal cells (NPCs) were obtained from donor livers rejected for transplantation or patients with primary or metastatic liver tumors undergoing liver resection at the Liver Cell Laboratory, Unit of Transplantation Surgery, Karolinska Institutet, Stockholm, Sweden. Only non‐tumor tissue from patients undergoing liver resection for primary or secondary liver malignancies was included. Clinical characteristics, causes of death, and donor types are summarized in Table S2. Formalin‐fixed, paraffin‐embedded (FFPE) liver samples from MASLD and MASH patients (*n* = 16) were obtained from the FLIS‐1 cohort [76]. Clinical characteristics and histological assessments are detailed in Table S3. Liver biopsies for immunoblotting were collected from patients with obesity undergoing Roux‐en‐Y gastric bypass (RYGB) surgery (*n* = 8) at Danderyd Hospital or Ersta Hospital, Stockholm, Sweden, and from normal weight donors (*n* = 4) undergoing liver transplantation. Clinical characteristics and causes of death are detailed in Tables S4 and S5, respectively. SVF from VAT and SAT samples were obtained from 20 patients with obesity undergoing RYGB surgery at Danderyd Hospital. None of the participants had a history of alcohol abuse, coagulopathy, chronic inflammatory diseases, or clinical signs of liver damage, nor had they undergone surgical intervention within six months before the study. Patients did not follow a specific diet before surgery. In six of these patients, VAT, SAT, and liver biopsies were used for FFPE. Clinical parameters are listed in Table S6. Longitudinal PBMCs and SAT biopsies were obtained from a subset of COCKTAIL study participants, where individuals with obesity underwent a very low‐calorie diet (VLED) with or without RYGB. In detail, participants followed a three‐week low‐energy diet (<1200 kcal/day) before being assigned to either the bariatric surgery group (RYGB followed by VLED, <800 kcal/day) or the diet‐only group (VLED) for six weeks. Patients were continuously monitored and advised to follow local dietary guidelines. Data up to week 20 are presented in this work. Clinical parameters and sample exclusion criteria are provided in Tables S7 and S8. PBMCs for flow cytometry and in vitro experiments were obtained from buffy coat blood collected from anonymous healthy donors at the Department of Transfusion Medicine, Karolinska Institutet. The studies were approved by the Regional Ethical Committee in Stockholm, Sweden, or Oslo, Norway, and all participants provided written informed consent before participation. Liver tissue from deceased organ donors was included in accordance with Sweden's organ transplantation law (1995:831), requiring adherence to the donor's prior written declaration and informed consent from next of kin.

### 4.2 Isolation of SVF From Adipose Tissue Biopsies

Adipose tissue was digested in Hank's Balanced Salt Solution (HBSS, Invitrogen) containing 1.28 mg/mL type II collagenase and 0.5% bovine serum albumin (BSA, Sigma). Tissue was minced into ∼1–2 mm fragments, transferred to 50 mL tubes with digestion buffer, and incubated at 37°C with shaking (200 rpm) for 20–30 min. Digestion was stopped with excess complete RPMI. After centrifugation (300 rpm, 5 min, 4°C), the adipocyte layer was discarded. The remaining suspension was filtered (70 µm), centrifuged (400×*g*, 5 min, 4°C), treated with red blood cell lysis buffer for 3 min, and washed in PBS. The final cell pellet, representing the SVF, was cryopreserved in heat‐inactivated fetal bovine serum (FBS) with 10% dimethyl sulfoxide (DMSO) and stored in liquid nitrogen.

### 4.3 Isolation of NPC From Human Livers

Liver non‐parenchymal cells (NPCs) were isolated using a three‐step collagenase perfusion protocol from livers obtained following partial or complete hepatectomy. Briefly, livers were perfused at 37°C with HBSS supplemented with ethylene glycol tetraacetic acid (EGTA; Sigma), followed by perfusion with either HBSS or Eagle's minimum essential medium (EMEM) supplemented with collagenase XI (Sigma). After enzymatic digestion, liver tissue was transferred into ice‐cold EMEM, mechanically dissociated into small fragments, and filtered through sterile gauze. Collagenase was removed by centrifugation, and cell pellets were washed twice with cold EMEM. Hepatocytes were separated by three consecutive centrifugations at 50×*g* for 5 min at 4°C. The NPC fraction was collected from the supernatant after the first centrifugation step, and cells were further isolated using Ficoll‐Paque (Cytiva) density gradient centrifugation. Isolated cells were cryopreserved in heat‐inactivated FBS containing 10% DMSO and stored in liquid nitrogen.

### 4.4 PBMCs and Monocyte Isolation

PBMCs were isolated from healthy donor buffy coats or heparin blood using Ficoll‐Paque (Cytiva) density gradient centrifugation in SepMate tubes (STEMCELL Technologies), following the manufacturer's protocol. Isolated PBMCs were either cryopreserved in 90% FBS with 10% DMSO or processed for monocyte purification. For primary monocyte purification, PBMCs were incubated with a negative selection microbead cocktail (Pan Monocyte Isolation Kit, Miltenyi Biotech) and isolated by magnetic‐activated cell sorting according to the manufacturer's instructions. Purified monocytes were either cryopreserved in 90% FBS and 10% DMSO in liquid nitrogen or used immediately for macrophage differentiation.

### 4.5 Monocyte Culture

Purified fresh or cryopreserved monocytes were seeded at a density of 1 × 10^6^ cells/mL and differentiated into macrophages by culturing with 50 ng/mL macrophage colony‐stimulating factor (M‐CSF, PeproTech) for 7 days. Cells were maintained in RPMI 1640 medium (Cytiva) supplemented with 10% heat‐inactivated FBS (Sigma‐Aldrich), 100 U/mL penicillin, and 100 µg/mL streptomycin (Cytiva) at 37°C and 5% CO_2_. Culture media were replaced, and fresh cytokines were added every 3 days. Where indicated, monocytes were differentiated into macrophages using 10 ng/mL granulocyte‐macrophage colony‐stimulating factor (GM‐CSF, PeproTech) instead of M‐CSF. Before experimental assays, macrophages were rinsed twice with ice‐cold PBS, incubated with 0.25% trypsin‐EDTA (Sigma‐Aldrich) for 15 min, and neutralized with complete RPMI. Cells were then rinsed with PBS and used in each assay as described in separate sections.

### 4.6 siRNA

Monocyte‐derived macrophages were transfected with small interfering RNAs (siRNAs) using Lipofectamine RNAiMAX (Invitrogen). siRNA–lipid complexes were prepared in Opti‐MEM (Gibco) according to the manufacturer's instructions and applied to cells at a final siRNA concentration of 50 nM. Cells were incubated with the transfection mixture overnight, after which the medium was replaced with complete RPMI. Following medium replacement, cells were allowed to recover for 24 h. At 48 h post‐transfection, cells were either harvested directly or subjected to downstream analysis or treatments. For metabolic syndrome‐like (MetS‐like) stimulation or targeted protein analyses, cells were further incubated for an additional 24 h before collection. The Silencer Select siRNAs used in this study (Invitrogen) are listed in Table S9.

### 4.7 CRISPR–Cas9 RNP Nucleofection

Lyophilized CRISPR RNA (crRNA) and trans‐activating CRISPR RNA (tracrRNA) were resuspended in nuclease‐free buffer according to the manufacturer's instructions (IDT). Equal molar amounts of crRNA and tracrRNA (100 µM each) were mixed and incubated at 95°C for 5 min, followed by cooling at room temperature for 10–15 min to allow formation of a 50 µM guide RNA duplex. The guide RNA duplex was subsequently incubated with TrueCut Cas9 Protein v2 (5 µg/µL; 31 µM) at an approximately 2.4:1 RNA:Cas9 molar ratio for 15 min at room temperature to form ribonucleoprotein (RNP) complexes at a final concentration of 12.5 µM. RNPs were prepared immediately before use. For ADAR1 targeting, two independent crRNAs targeting distinct regions of ADAR1 were used; each crRNA was assembled into RNPs separately with Cas9, and the resulting RNPs were combined in equal volumes before nucleofection to enhance editing efficiency. Two nontargeting crRNAs were used as negative controls. crRNA Sequences are listed in Table S9.

Isolated human monocytes were counted, and 1 × 10^6^ cells per nucleofection reaction were pelleted by centrifugation at 300×*g* for 5 min and washed once with PBS. After complete removal of the supernatant, cells were resuspended in 20 µL per reaction of room‐temperature Lonza P3 nucleofection buffer. The cell suspension was gently mixed with a total of 8 µL per reaction of RNP complexes and transferred to a 16‐well Nucleocuvette Strip for nucleofection using the Lonza 4D‐Nucleofector system (program CM‐137; Lonza). Immediately following nucleofection, 80 µL of prewarmed culture medium was added to each well, and cells were rested for 15–30 min at 37°C in a humidified incubator with 5% CO_2_. Cells were subsequently transferred to flat‐bottom 96‐well plates for monocyte‐to‐macrophage differentiation or used for spheroid seeding, as indicated.

### 4.8 Cell Viability

CellTiter Glo 2.0 A Luminescent Cell Viability Assay Kit (Promega) was used to assess cell viability in transfected MDMs by quantifying ATP levels using the Tecan Infinite M200 plate reader.

### 4.9 Primary Human Hepatic Spheroid Formation

Cryopreserved primary human hepatocytes (PHH) and hepatic stellate cells (HSC) were obtained from BioIVT with donor consent. HSCs were expanded in Dulbecco's Modified Eagle Medium (DMEM) supplemented with 10% heat‐inactivated FBS, 100 U/mL penicillin, and 0.1 mg/mL streptomycin for three passages. Monocytes were thawed, washed once with PBS, and used immediately or nucleofected as described above. Spheroids were generated as previously described [77, 78, 79], with minor modifications. Briefly, cells were seeded into 96‐well ultra‐low attachment plates (BIOFLOAT, faCellitate) in 100 µL William's E medium (PAN Biotech) supplemented with 5.5 mM glucose (Sigma‐Aldrich), 1 nM insulin (Gibco), 100 nM dexamethasone (Sigma), 5.5 mg/L transferrin (Sigma), 6.7 µg/L sodium selenite (Sigma), 1% GlutaMAX, 100 U/mL penicillin, 0.1 mg/mL streptomycin, and 10% heat‐inactivated FBS. Spheroid compositions included: PHH alone (2000 cells/well), PHH+HSC (1,950 PHH + 50 HSC), and PHH+HSC+monocytes (1750 PHH + 50 HSC + 200 monocytes). FBS was gradually phased out during the first 7 days of aggregation. At day 7, spheroids were either harvested or maintained for an additional 7 days under MetS‐like or vehicle control conditions. MetS‐like media contained 0.8% albumin‐conjugated free fatty acid mix (240 µM palmitic acid, 240 µM oleic acid), 11 mM glucose, 100 nM insulin, and 100 µM fructose. Vehicle control contained 0.8% albumin in serum‐free seeding media. Media were refreshed every other day by replacing 50% of the well volume.

### 4.10 Flow Cytometry

Human tissue and blood samples were phenotyped using a BD FACSymphony A3 or A5 flow cytometer. Cryopreserved cells were thawed in complete RPMI, washed with PBS, and incubated with Zombie Aqua viability dye (BioLegend) for 10 min. Then, cells were resuspended in FACS buffer (PBS + 2% FBS + 4 mM EDTA) and incubated with Fc‐block (BD Pharmingen) for 10 min. Extracellular staining was performed for 30 min using fluorophore‐conjugated antibodies, followed by fixation using the Foxp3/transcription factor staining kit (eBioscience) for 1 h at 4°C. Intracellular staining was carried out overnight in permeabilization buffer containing 2% mouse (eBioscience) and 2% goat serum (Cell Signaling). For in vitro experiments, detached cells were processed using the same protocol and acquired on a BD FACSymphony A3 or BD Fortessa. All staining and centrifugation steps were conducted in the dark at 4°C. Data were analyzed using FlowJo (v10.10). The list of antibodies and panels used for each analysis is detailed in Tables S10–S16.

### 4.11 Western Blot

Cells, spheroids, or tissues were lysed in RIPA buffer (Thermo Fisher Scientific) supplemented with protease and phosphatase inhibitors (Roche). Before lysis, cells and spheroids were thoroughly washed with PBS. Liver biopsies were homogenized using 5 mm stainless steel beads (Qiagen) in a TissueLyser LT, followed by gentle rotation at 4°C for 1 h. Lysates were cleared by centrifugation at 12,000×*g* for 15 min at 4°C. Protein concentrations were quantified using the Pierce BCA protein assay kit (Thermo Fisher Scientific). For Western blot analysis, lysates were heated at 95°C for 5 min, separated on NuPAGE 4%–12% Bis‐Tris gels (Invitrogen) at 140 V for 1–2 h, and transferred onto nitrocellulose membranes (BioRad) at 2.5A for 7 min. Membranes were blocked with 5% milk (Merck) in Tris‐buffered saline with 0.5% Tween‐20 (Merck) (TBST) for 1 h at room temperature (RT), then incubated overnight at 4°C with primary antibodies diluted in TBST containing 5% BSA and 0.1% sodium azide (Sigma‐Aldrich). Detection was performed using HRP‐conjugated secondary antibodies (Abcam, Agilent Technologies) and visualized using a Li‐Cor Odyssey or a Bio‐Rad imaging system. Band intensities were quantified using Li‐Cor Odyssey Imager or ImageJ software, respectively. Antibodies and dilutions are detailed in Table S17.

### 4.12 RT‐qPCR

Total RNA was extracted from cells using the Direct‐Zol RNA Micro Kit (Zymo Research) following the manufacturer's instructions. Reverse transcription was performed with the iScript reverse transcription reagent kit (Bio‐Rad) according to the manufacturer's protocol. Quantitative PCR (qPCR) for *ADAR* and *TBP* expression was performed using the SsoAdvanced Universal SYBR Green Supermix (Bio‐Rad) on the CFX96 Real‐Time PCR System (Bio‐Rad). Each reaction included 5 µL cDNA, 1 µL each of forward and reverse primer (1 µM), and 7 µL SYBR Green Supermix. Gene expression was normalized to *TBP* for monocytes and macrophages. Relative expression was calculated using the 2^−ΔΔCt^ method. Primers were purchased from Integrated DNA Technologies and are listed in Table S18.

### 4.13 Functional Assays in Human Macrophages

MDMs were incubated with 0.25 µg/mL pHrodo Green *E. coli* BioParticles (Invitrogen) to assess phagocytosis. For endocytosis, MDMs were treated with 50 µg/mL pHrodo Green Dextran (Invitrogen) and 50 µg/mL Dextran, Alexa Fluor 647, 10,000 MW (Invitrogen). BioParticles and Dextran molecules were incubated with the cells for 60 min at 37°C and 5% CO_2_. For the proteolysis assay, 25 µg/mL DQ Red BSA (Invitrogen) was added to MDMs for 60 min, followed by the addition of 75 nM LysoSensor Green DND‐189 (Invitrogen) for the last 30 min of the incubation period. Following the incubation, cells were lifted as described above and resuspended in FACS buffer. For lipid accumulation, cells were lifted, fixed, and stained with LipidTOX Red (1:500, Invitrogen) for 30 min at room temperature in the dark. Cells were then analyzed using a BD FACSymphony A3 flow cytometer. Gating was performed based on forward scatter (FSC)/side scatter (SSC) properties, and doublets were removed. For the phagocytosis assay, the frequency and/or gMFI of pHrodo Green *E. coli* BioParticles was measured. In the endocytosis assay, the gMFI of pHrodo Green Dextran and its ratio to the gMFI of Dextran, Alexa Fluor 647 were assessed. For the proteolysis assay, the gMFI ratio of DQ Red BSA to LysoSensor Green was determined.

### 4.14 Efferocytosis Assay

Jurkat human T lymphocytes were cultured in DMEM, supplemented with 10% heat‐inactivated FBS, 100 U/mL penicillin, and 0.1 mg/mL streptomycin. For labelling, cells were incubated with 1 µM Calcein Green AM (Invitrogen) in PBS for 30 min at 37°C and 5% CO_2_, then washed twice with complete DMEM. Apoptosis was induced by treatment with 2 µM staurosporine (Sigma‐Aldrich) for 2 h followed by washing and resuspension in complete RPMI. Apoptotic Jurkat cells were co‐cultured with MDMs at a 5:1 ratio for 60 min. Subsequently, cells were washed three times with ice‐cold PBS, lifted, and resuspended in FACS buffer. Flow cytometry was performed on a BD FACSymphony A3. Gating was performed based on FSC and SSC properties to identify singlets. Efferocytosis was assessed by quantifying the frequency of macrophages positive for Calcein‐AM‐labeled apoptotic cells.

### 4.15 Dead Cell and Apoptosis Assay

Cell death in ADAR1‐silenced MDMs was evaluated using the Dead Cell Apoptosis Kit with Annexin V Alexa Fluor 488 and Propidium Iodide (Invitrogen) according to the manufacturer's instructions. Briefly, cells were detached, washed with PBS, and stained with Annexin V and Propidium Iodide. Stained cells were then analyzed using a BD FACSymphony A3 flow cytometer. Gating was performed to exclude debris and doublets, and populations of live (Annexin V^–^/PI^–^), early apoptotic (Annexin V^+^/PI^–^), and late apoptotic/necrotic (Annexin V^+^/PI^+^) cells were quantified.

### 4.16 Fluorescence Immunocytochemistry

Monocytes were differentiated into macrophages in 96‐well black TC‐treated plates (Corning) or on coverslips. After siRNA treatments, live cells were incubated with 200 nM LysoTracker Red DND‐99 (Invitrogen) and 75 nM LysoSensor Green DND‐189 at 37°C and 5% CO_2_ for 30 min, rinsed twice with PBS, and resuspended in Live Cell Imaging Solution (Invitrogen) for imaging. For ADAR1 detection, cells were fixed with 4% methanol‐free formaldehyde (Thermo Scientific) for 15 min, permeabilized with 0.2% Tween 20 and 0.1% Triton X‐100 for 15 min, and blocked with 3% BSA and 0.2% Tween 20 for 1 h. Cells were then incubated overnight at 4°C with an ADAR1‐specific primary antibody (Atlas Antibody), followed by a 2 h incubation with a goat anti‐rabbit Alexa Fluor 555‐conjugated secondary antibody (1:500, Invitrogen) and DAPI staining (Sigma‐Aldrich) for 5 min. Alternatively, fixed MDMs were incubated for two hours with an anti‐rabbit Alexa Fluor 647‐conjugated ADAR1 antibody (1:100, Cell Signaling). For the lipid accumulation assessment, fixed MDMs were treated with LipidTOX Green (1:1000, Invitrogen) for 30 min at RT in the dark. Images were acquired using a Zeiss inverted fluorescence microscope (ZEN 3.0) and analyzed using QuPath (version 0.5.1) [80]. Cell segmentation was performed using StarDist (model dsb2018_heavy_augment) [81]. Object classifiers were assigned for LysoTracker^+^ (Cell Max intensity), ADAR1^+^ (Nuclear Mean), and LipidTOX^+^ (Cell Mean). Classifier results were exported as cell annotations and further analyzed in R.

### 4.17 Immunofluorescence on Paraffin‐Embedded Tissues

Liver, SAT, and VAT biopsies were fixed overnight in 10% formalin (Sigma‐Aldrich) at 4°C, embedded in paraffin, and sectioned at 5 µm thickness. Sections were deparaffinized with two 5 min incubations in 100% xylene, followed by sequential washes in 100%, 95%, and 70% ethanol, then rinsed in distilled water. Antigen retrieval was performed by boiling the slides in sodium citrate buffer (0.02% Tween 20, pH 6.0) for 10 min in a pressure cooker, followed by cooling at RT for 1–2 h. Permeabilization and blocking were carried out using Background Buster (Innovex) containing 0.1% Triton X‐100 (BBT) for 30 min. Slides were incubated overnight at 4°C with primary antibodies (ADAR1, CD68, listed in Table S17) diluted in BBT, followed by 1‐hour incubation with fluorescent secondary antibodies (goat anti‐rabbit AF555, goat anti‐mouse AF647, 1:500, Invitrogen) at RT. Nuclei were stained with DAPI (Sigma‐Aldrich) for 5 min before mounting. Images were acquired at 20× using a Zeiss inverted fluorescence microscope using Zen software (version 3.0) and analyzed using QuPath (0.5.1) [80]. Individual cells were segmented using Stardist (model dsb2018_heavy_augment) [81]. Cell classifiers for CD68^+^ (Cell Mean) and ADAR1^+^ (Cell Mean) expression were optimized per donor for paired analysis in SAT, VAT, and liver from patients with obesity undergoing bariatric surgery. For FFPE MASLD/MASH livers, cell classifiers for CD68^+^ (Cell Mean) and ADAR1^+^ (Nuclear Median) were assigned per experimental batch. ADAR1^+^ nuclear fluorescent intensity was normalized to nucleus size. Classifier results were exported and analyzed in R. For each biological replicate of each tissue, three to six random areas per tissue section were selected as technical replicates.

### 4.18 Lipid Accumulation Assessment on Whole Mount Hepatic Spheroids

Hepatic spheroids were fixed in 4% methanol‐free formaldehyde, stained with Hoechst 33342 (Invitrogen) and LipidTOX Deep Red (Invitrogen), and imaged on a Nikon A1R confocal microscope at 20× magnification. For each spheroid, 7–12 Z‐planes were acquired. Z‐stacks were imported into Fiji/ImageJ, and a SUM Z‐projection was generated for the LipidTOX channel. Individual spheroids were manually outlined as regions of interest (ROIs). Within each ROI, a fixed intensity threshold per donor was applied to identify LipidTOX‐positive regions, and the area of LipidTOX signal was measured.

### 4.19 Biochemical Assessment of Intracellular Lipids in Hepatic Spheroids

Hepatic spheroids were washed with PBS and transferred individually into wells of a 96‐well flat black, clear‐bottom plate (Corning) containing 200 µL of 0.25% trypsin‐EDTA (phenol red–free; Gibco) supplemented with 2.5 µg/ml of collagenase IV. Spheroids were incubated at 37°C for 30 min to facilitate dissociation. Subsequently, 5 µL of AdipoRed reagent (Lonza) was added to each well and mixed thoroughly in the dark until the spheroids were no longer visible. After 10 min of incubation at room temperature, fluorescence was measured using a Tecan Infinite F500 plate reader with excitation/emission settings of 485/590 nm. Relative fluorescence units (RFU) from wells containing AdipoRed and digestion media but no spheroids were subtracted from sample readings to correct for background.

### 4.20 RNA‐seq Library Preparation and Analysis

For siADAR1, siADAR1‐p150, and negative‐control silenced macrophages, cells were harvested 48 h post‐transfection. For CRISPR ADAR1^p110‐/p150high^ MDMs, cells were harvested after 7 days of differentiation. Total RNA was extracted as described above. Sequencing libraries were prepared using the TruSeq Stranded mRNA Kit (Illumina). The sequencing library was then analyzed using the NovaSeq Plus platform, employing the PE150 model at a sequencing depth of 50–70 million paired‐end reads per sample.

### 4.21 RNA‐seq Analysis

Alignment, variant calling, and quality control were performed using the RNA‐seq pipeline within the bcbio‐nextgen framework (1.2.9) [82]. Briefly, RNA‐seq data were aligned to the hg38 human reference genome using the STAR [83]. Quality control was performed with MultiQC [84], and transcript quantification was conducted using Salmon [85]. Gene‐level counts were generated from the Salmon counts using the tximport package for downstream analysis. Differential expression was calculated using DESeq2 (1.54.1) [86], with a significance threshold set at adjusted *p*‐values < 0.05. Visualization of data, including PCA, heatmaps, volcano plots, and correlation plots, was conducted using the pheatmap (1.0.13) and ggplot2 (3.5.2) packages in R.

### 4.22 Exon Level Analysis

RNA‐seq reads were processed using bcbio‐nextgen (1.2.9) with the DEXSeq expression caller [87], which performs read trimming, alignment to the reference genome (hg38), and quantification of reads in nonoverlapping bins per exon. Differential exon usage between Control and p110^−^/p150^high^ knockout samples was tested using DEXSeq (1.54.1), modeling donor as a covariate. Library size normalization was performed via size factor estimation, and bin‐wise dispersions were calculated. Differential usage was assessed using a negative binomial Wald test, and log2 fold changes were computed for each bin. For visualization, bin‐level results were aggregated to the exon level by taking a weighted mean per exon based on bin length. These data were used to generate heatmaps and scaled exon usage plots in R using ggplot2 (3.5.2).

For the monocyte‐to‐macrophage differentiation dataset (GSE147309) [88], which contained a single donor across multiple differentiation days, normalized counts per bin were summed to obtain summarized exon‐level counts. Summarized counts were then used to generate a heatmap in R using pheatmap (1.0.13).

### 4.23 A‐to‐I RNA Editing Analysis

RNA‐seq reads were aligned to the human reference genome (GRCh38) using STAR (version 2.7.5b) with customized parameters to optimize splice junction detection and alignment quality. Postalignment processing was performed using SAMtools (1.10), Picard (2.23.3), and RSeQC (3.0.1) to remove multimapped reads and PCR duplicates. To ensure alignment quality, junction‐spanning reads were split into supplementary alignments, followed by base quality score recalibration using known single‐nucleotide polymorphisms (SNPs) sites from dbSNP, 1000 Genomes, and exome variant server (EVS) databases. The resulting BAM files were then filtered to retain reads mapped to Alu repeat regions, as defined by RepeatMasker annotations from the UCSC Genome Browser. To minimize artifacts from random hexamer priming, the first five bases of each read were soft‐clipped using bamUtil trimBAM. Variant calling was performed using BCFtools mpileup, requiring a minimum mapping quality threshold of 20 and a minimum base quality threshold of 25. To exclude known genomic variants, variants overlapping known SNPs in the dbSNP, 1000 Genomes, and EVS databases were removed. The AEI was calculated as the percentage of the number of A‐to‐G and T‐to‐C mismatches to the sum of matches (A–A and T–T) and mismatches (A‐to‐G and T‐to‐C) [89]. For over‐representation analysis, genes with more than 50 detected RNA editing events across samples were selected. Editing counts were normalized to sequencing depth per sample. Normalized per‐gene editing counts were analyzed using linear mixed‐effects models with siRNA treatment as a fixed effect and donor as a random effect. Bar plots were generated in GraphPad Prism (10.6.1). Circos plots, paired dot plots, Venn diagrams, and heatmaps were visualized using the circlize (0.4.17), ggplot2 (3.5.2), and pheatmap (1.0.13) packages in R.

### 4.24 TMT Proteomics Sample Preparation

For siADAR1, siADAR1‐p150, and negative‐control silenced macrophages, cells were harvested 48 h post‐transfection. Cells were washed with ice‐cold PBS, detached, pelleted at 4°C, and stored at −80°C. Cell pellets were lysed in 4% SDS lysis buffer and prepared for mass spectrometry analysis using a modified SP3 protein cleanup and digestion protocol. Peptides were labeled with TMTpro 16‐plex reagents according to the manufacturer's instructions (Thermo Fisher Scientific). Labeled peptides were cleaned by strong cation exchange–based solid‐phase extraction and fractionated by immobilized pH gradient isoelectric focusing (IPG‐IEF, pH 3–10) as described previously [90]. Peptide fractions were analyzed by online LC–MS/MS using dynamic gradients on a Vanquish Neo UHPLC system coupled to a Q Exactive Exploris mass spectrometer (Thermo Fisher Scientific). Raw data were processed using Proteome Discoverer (v2.4) with the Sequest HT search engine and Percolator against the UniProt human reference proteome, applying a 1% false discovery rate cutoff.

### 4.25 TMT Proteomics Analysis

Protein abundance values were normalized to the total protein abundance per sample and log2‐transformed. Proteins lacking complete quantitative data across samples were excluded. Differential protein abundance was assessed using the limma package (3.64.3) in R [91], fitting a linear model with treatment as the main effect and donor included as a covariate. Statistical significance was defined using an FDR threshold of <0.1, combined with an absolute fold change >10%. Where indicated, a more permissive threshold of nominal p‐value < 0.05 and absolute fold change > 7% was applied. Overrepresentation analysis was performed separately for upregulated and downregulated DEPs for each ADAR1 silencing condition relative to the control. Data analysis and visualization, including PCA, heatmaps, volcano plots, Venn diagrams, and dot plots, were conducted in R using the pheatmap (1.0.13) and ggplot2 (3.5.2) packages.

### 4.26 Functional Enrichment and Pathway Analysis

Overrepresentation analyses were performed using WebGestalt [92] for DEGs, highly edited genes, and DEPs. Analyses were conducted separately for upregulated and downregulated features using Gene Ontology, KEGG, Reactome, and WikiPathways, as appropriate. Pathways with a false discovery rate <0.05, calculated using the Benjamini–Hochberg procedure, were considered significant.

### 4.27 Integrative Genomics Viewer Browser

For visualization of RNA editing sites in the 3'‐UTR of *CPT1A* across samples, VCF files containing detected RNA editing sites within Alu elements were examined using the IGV browser [93].

### 4.28 Analysis of Public RNA‐seq Datasets

ADAR1 mRNA expression levels were assessed using publicly available RNA‐seq datasets from the Gene Expression Omnibus (GEO) [94]. Transcripts per million (TPM) values were downloaded from the NCBI‐generated RNA‐seq normalized count data. Counts per million (CPM) values were obtained when indicated by the submitter. For a subset of these datasets, the Alu editing index was also assessed to evaluate potential changes in ADAR1 activity. Datasets used in this analysis are grouped by the tissue of origin and are listed as follows: Human monocytes/monocyte‐derived macrophages: PRJNA449980 [95], GSE147310 [88], and GSE165152 [96]. SAT biopsies in individuals with normal weight and obesity: GSE205668 [97], GSE162653 [98], GSE244118 [99], GSE156906 [100], and GSE159924 [101]. Liver biopsies in obesity and MASLD: GSE239422 [102], GSE167523 [103], GSE193066 [104], GSE130970 [105], PRJNA512027 [106], GSE213621 [107], and GSE135251 [108]. SAT biopsies in caloric intervention: GSE168705 [109], GSE274137 [110], GSE106289 [60], and GSE95640 [111].

### 4.29 Statistics

Statistical analyses were performed using GraphPad Prism software (10.6.1) or R (4.5.1). Data normality was assessed using D'Agostino–Pearson, Shapiro–Wilk, and Kolmogorov–Smirnov tests where applicable. For normally distributed data, appropriate parametric tests were applied, including unpaired or paired Student's *t‐*tests, one‐ or two‐way analysis of variance (ANOVA), or mixed‐effects models, followed by Tukey's post hoc multiple comparisons test where applicable. For nonnormally distributed data, nonparametric tests were used, including the Mann–Whitney *U* test or Wilcoxon matched‐pairs signed‐rank test, as appropriate. A one‐sided Wilcoxon signed‐rank test was used to assess differences in RNA editing indices between control and ADAR1‐silencing conditions, based on the a priori hypothesis that ADAR1 depletion would lead to a reduction in editing activity. Correlations were assessed using Spearman's correlation coefficient. Statistical significance was defined as *p* < 0.05, with the following notation: *p < 0.05, **p < 0.01, and ***p < 0.001.

## Author Contributions

**Conceptualization**: Achilleas Fardellas, Niklas K. Björkström, and Myriam Aouadi. **Methodology**: Achilleas Fardellas and Niklas K. Björkström. **Software**: Achilleas Fardellas and Ping Chen. **Investigation**: Achilleas Fardellas, Emelie Barreby, Madara Brice, Sebastian Nock, Jules Russick, Ana Vankova, Charlotte Edberg, Ida Robertsen, Jens K. Hertel, Per Stål, Gunnar Mellgren, Cecilia Karlsson, Jøran S. Hjelmesaeth, Hannes Hagström, Erik Näslund, Johan Fernø, and Cecilia Morgantini. **Resources**: Ida Robertsen, Jens K. Hertel, Per Stål, Gunnar Mellgren, Cecilia Karlsson, Jøran S. Hjelmesaeth, Hannes Hagström, Erik Näslund, Johan Fernø, and Cecilia Morgantini. **Data curation**: Achilleas Fardellas, Charlotte Edberg, Ida Robertsen, Jens K. Hertel, Per Stål, Gunnar Mellgren, Cecilia Karlsson, Jøran S. Hjelmesaeth, Hannes Hagström, Erik Näslund, and Cecilia Morgantini. **Visualization, supervision**, **funding acquisition, writing – original draft**: Achilleas Fardellas and Niklas K. Björkström. **Writing – review and editing**: All authors.

## Funding

This work was supported by the European Research Council (ERC) under the European Union's Horizon 2020 research and innovation program (grants agreement no. 948692, 864788, 101170408), the Swedish Research Council (2020‐06250), the Swedish Cancer Society, the Swedish Foundation for Strategic Research, Knut and Alice Wallenberg Foundation, the Center for Innovative Medicine at Karolinska Institutet, Region Stockholm, the NovoNordisk Foundation, Swedish Society for Medical Research (SSMF), Swedish Society of Medicine, Karolinska Institutet, Research Council of Finland, Robert Bosch Foundation, Stuttgart, Germany, the Western Norway Regional Health Authority and Haukeland University Hospital. The COCKTAIL study was funded by the three collaborators: (1) Vestfold Hospital Trust, Tønsberg, Norway; (2) School of Pharmacy, University of Oslo, Oslo, Norway, and (3) AstraZeneca Gothenburg, Sweden.

## Conflicts of Interest

Cecilia Karlsson is employed and a shareholder at AstraZeneca AB. Volker M. Lauschke is cofounder and shareholder of HepaPredict AB, as well as the co‐founder and shareholder of Shanghai Biotechnology Ltd. The remaining authors declare no conflicts of interest.

## Declaration of Generative AI and AI‐Technologies in the Writing Process

During the preparation of this work, the authors used ChatGPT (OpenAI) to enhance the clarity and readability of the text. All content generated with the tool was subsequently reviewed and edited by the authors, who take full responsibility for the final content of the publication.

## List of Auxiliary Supplementary Materials

Table S1. List of RNA editing events per gene and pathway analysis.

Table S2. List of DEGs for siADAR1, siADAR1‐p150, and overlapping and pathway analysis for each comparison.

Table S3. List of DEPs for siADAR1 and siADAR1‐p150 and pathway analysis for each comparison.

Table S4. List of DEGs for ADAR1^p110‐/p150high^ and pathway analysis.

## Supporting information




**Supporting File 1**: eji70189‐sup‐0001‐SuppMat.pdf.

## Data Availability

The raw sequencing data from ADAR1‐silenced and ADAR1^p110‐/p150high^ human monocyte‐derived macrophages have been deposited in the National Center for Biotechnology Information (NCBI) GEO under accession numbers GSE307402 and GSE319320. The mass spectrometry proteomics data have been deposited with the ProteomeXchange Consortium [112] via the PRIDE partner repository [113] under dataset identifier PXD073582. Auxiliary Data Tables 1–4 are deposited in Zenodo (DOI:10.5281/zenodo.18609922). All code supporting this study is available from the corresponding author upon reasonable request.
