# Supplementary material for: ADAR1 Controls Macrophage Scavenging and Lipid‐Buffering Programs in Metabolic Tissues
Source: Eur J Immunol. 2026 Apr 26;56:e70189. doi: 10.1002/eji.70189 (PMC13111732; doi:10.1002/eji.70189)
Supplement: Supplementary file 1 — Supporting File 1: eji70189‐sup‐0001‐SuppMat.pdf. [file EJI-56-e70189-s001.pdf]

Supplementary Materials for

ADAR1 controls macrophage scavenging and lipid-buffering programs in metabolic tissues

Achilleas Fardellas, Emelie Barreby, Madara Brice, Sebastian Nock, Jules Russick, Ana Vankova, Charlotte Edberg, Ida Robertsen, Jens K. Hertel, Per Stål, Gunnar Mellgren, Cecilia Karlsson, Jøran S. Hjelmesaeth, Hannes Hagström, Erik Näslund, Volker M. Lauschke, Johan Fernø, Ping Chen, Cecilia Morgantini, Myriam Aouadi, Niklas K. Björkström

Fig. S1. ADAR1 is enriched in human macrophages *ex vivo*.
Fig. S2 ADAR1 protein levels increase during monocyte-to-macrophage differentiation *in vitro* by selective usage of an alternative TSS.
Fig. S3. Effects of ADAR1 silencing on macrophage viability and RNA editing.
Fig. S4. RNA-sequencing analysis of ADAR1 silencing in human macrophages.
Fig. S5. Global TMT proteomics analysis of ADAR1 silencing in human macrophages.
Fig. S6. Effects of ADAR1 silencing on phagocytosis and chloroquine responses in macrophages.
Fig. S7. Delineating macrophage subpopulations in SAT, VAT and liver biopsies.
Fig. S8. Regulation of ADAR1 in MASLD, establishment of organotypic spheroid model, and CRISPR KO ADAR1 in human macrophages.
Fig. S9. Regulation of ADAR1 in adipose tissue and peripheral immune cells in response to weight-loss.

Table S1. Cross-referenced annotation of CD206^{+} adipose tissue macrophages (ATM) subsets from single-cell and single nucleus RNA-seq studies.
Table S2. Patient list of liver NPC samples used for flow cytometry.
Table S3. Patient list of liver FFPE biopsies used for immunofluorescence.
Table S4. Clinical information of liver biopsies from normal weight individuals used for Western blot analysis.
Table S5. Clinical information of liver biopsies from patients with obesity used for Western blot analysis.

Table S6. Clinical information of liver, VAT and SAT biopsies from patients with obesity used for flow cytometry and immunofluorescence analyses.

Table S7. Clinical information and sample inclusion/exclusion of SAT SVF samples used for flow cytometry in the COCKTAIL study, grouped by visit.

Table S8. Clinical information and sample exclusion of PBMCs used for flow cytometry in the COCKTAIL study, grouped by visit.

Table S9. List of siRNA & crRNAs used in human MDMs.

Table S10. Flow cytometry panel for PBMCs from healthy human buffy coat donors.

Table S11. Flow cytometry panels for liver NPCs isolated from donor livers rejected for transplantation.

Table S12. Flow cytometry panel for liver NPCs isolated from donor livers rejected for transplantation or from patients undergoing resection.

Table S13. Flow cytometry panel for SAT and VAT SVF cells from patients with obesity and patients participating in the COCKTAIL study.

Table S14. Complementary flow cytometry panel for SAT and VAT SVF cells from patients with obesity.

Table S15. Flow cytometry panel for PBMCs from patients participating in the COCKTAIL study.

Table S16. List of antibodies used for flow cytometry in human MDMs.

Table S17. List of antibodies used for Western blot, IF and IHC.

Table S18. List of primers used for qPCR.

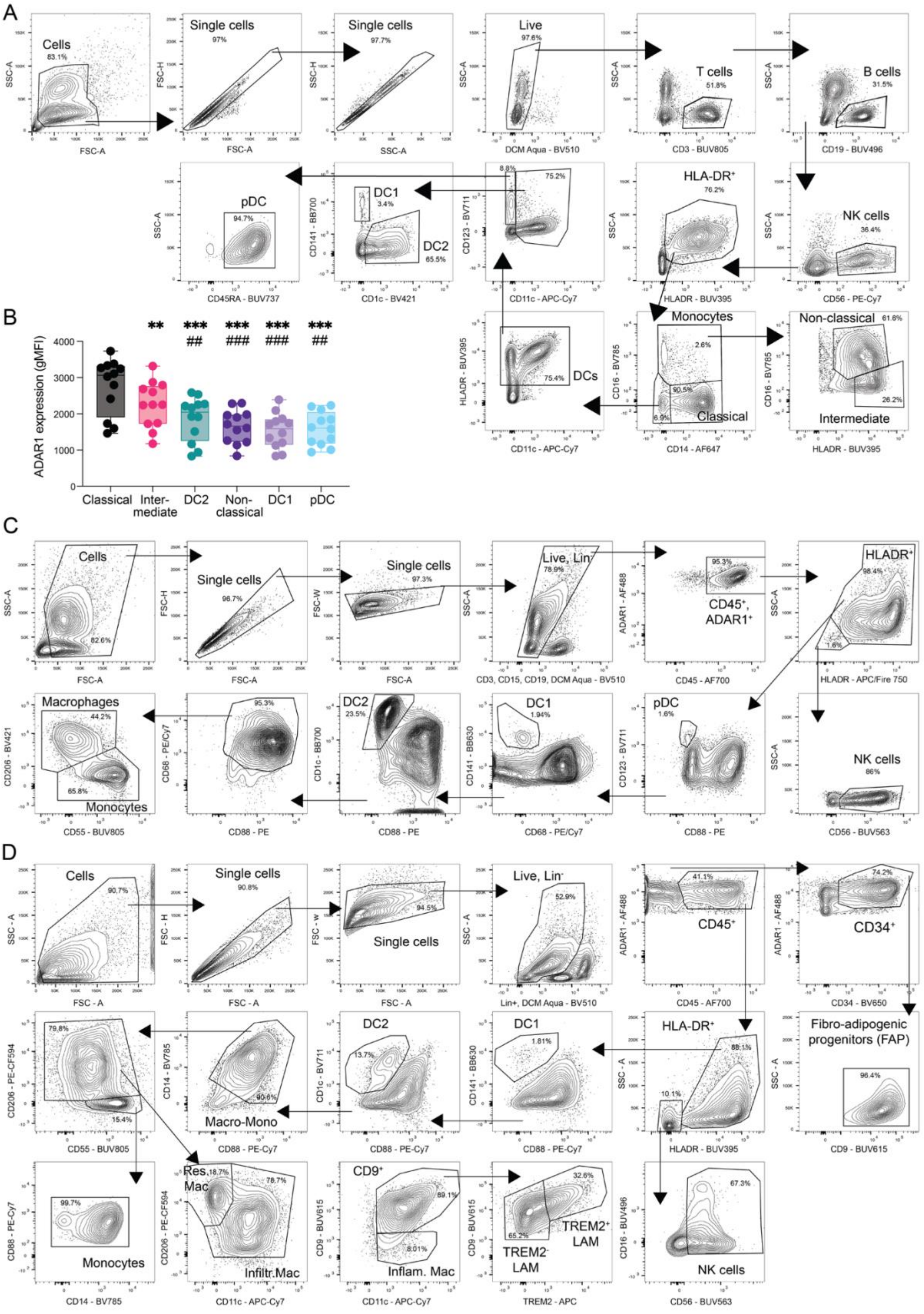

Fig. S1. ADAR1 is enriched in human macrophages *ex vivo*. (**A**) Representative flow cytometry gating strategy for PBMCs isolated from healthy donor buffy coats. (**B**) Box-and-

whisker plots showing ADAR1 expression (gMFI) in monocyte and dendritic cell subsets within PBMCs isolated from healthy buffy coats. Statistical annotations indicate comparisons relative to classical (*) or intermediate monocytes. (**C**) Representative flow cytometry gating strategy for NPCs isolated from perfused human livers. (**D**) Representative flow cytometry gating strategy for SVFs isolated from SAT or VAT biopsies from patients with obesity undergoing bariatric surgery. Box-and-whisker plots display min-max range, interquartile range (25th-75th percentiles) and median. Statistical significance was determined by ANOVA followed by Tukey's multiple comparisons test (**B**), *P < 0.05, **P < 0.01, and ***P < 0.001.

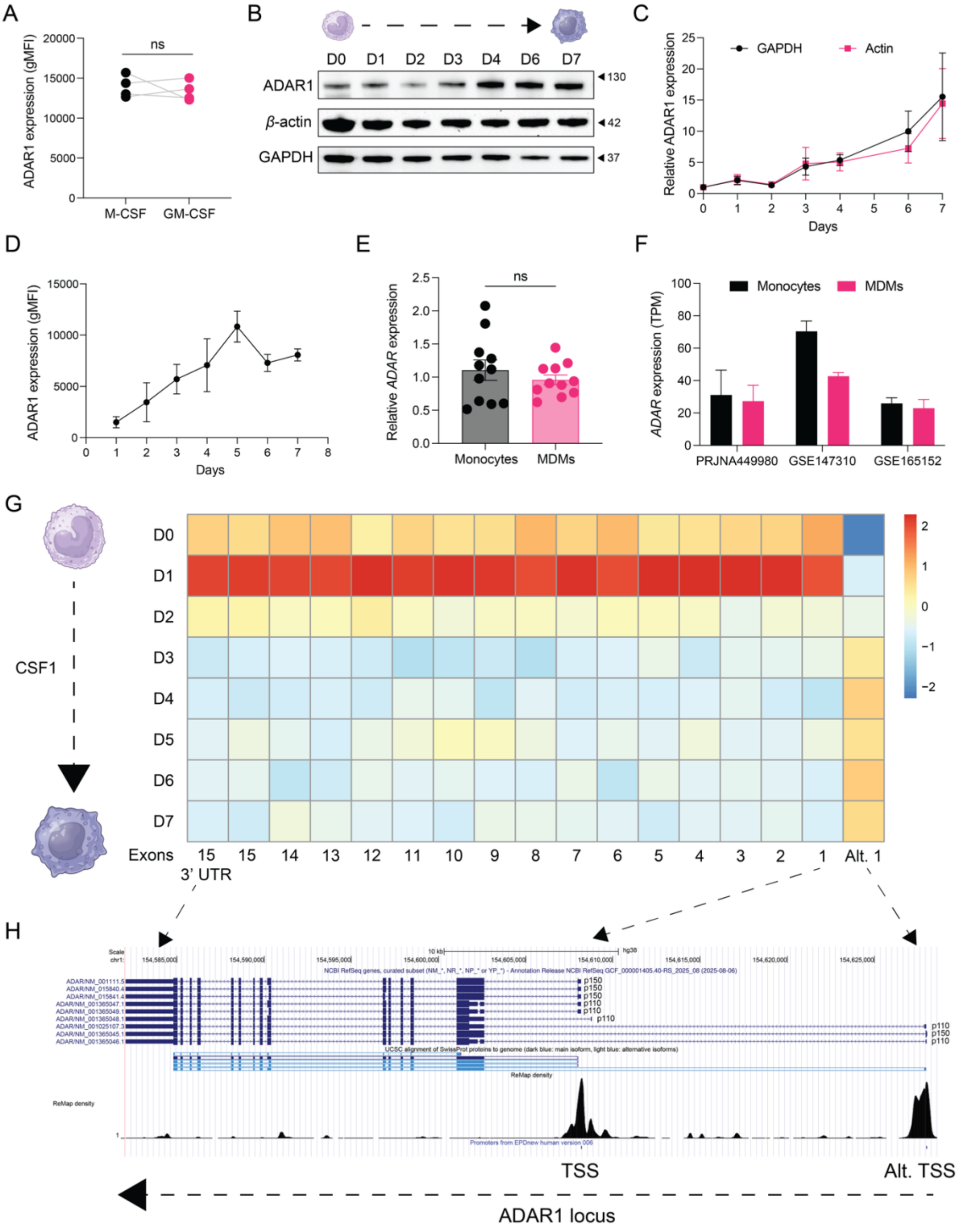

Fig. S2 ADAR1 protein levels increase during monocyte-to-macrophage differentiation *in vitro* by selective usage of an alternative TSS. (**A**) Paired dot plots of ADAR1 expression (gMFI) in human MDMs differentiated using M-CSF or GM-CSF from matched donors (n = 4). (**B & C**) Representative Western blot and quantification of ADAR1 protein expression

during monocyte-to-macrophage differentiation from two independent experiments (n = 5). *β*-actin or GAPDH was used as loading controls. Due to low basal ADAR1 abundance, double the total protein amount was loaded for monocytes to enable accurate protein detection and quantification. (**D**) Time-course flow cytometry analysis of ADAR1 expression (gMFI) during monocyte-to-macrophage differentiation from two independent experiments (n = 6). (**E**) Quantitative real-time PCR (qPCR) analysis of *ADAR* mRNA expression, with *TBP* used as a housekeeping control from three independent experiments (n = 11). (**F**) Quantification of *ADAR* mRNA expression (TPM) in three independent RNA-seq datasets comparing human monocytes and M-CSF-differentiated MDMs. (**G**) Exon-level of ADAR1 during monocyte-to-macrophage differentiation over time (n = 1; GSE147309). Scaled heatmap showing summarized, normalized exon-level counts of each exon at each indicated time point. DEXSeq bin-level results were aggregated to the exon level. (**H**) Genome browser view of the ADAR1 locus, showing transcript annotation, Uniprot features, RepMap density, and promoter information from EPDnew. Data in bar and line plots are presented as mean ± SEM. Box-and-whisker plots display min-max range, interquartile range (25th-75th percentiles) and median. Statistical significance was determined by ratio paired t-test (**A**, **E**).

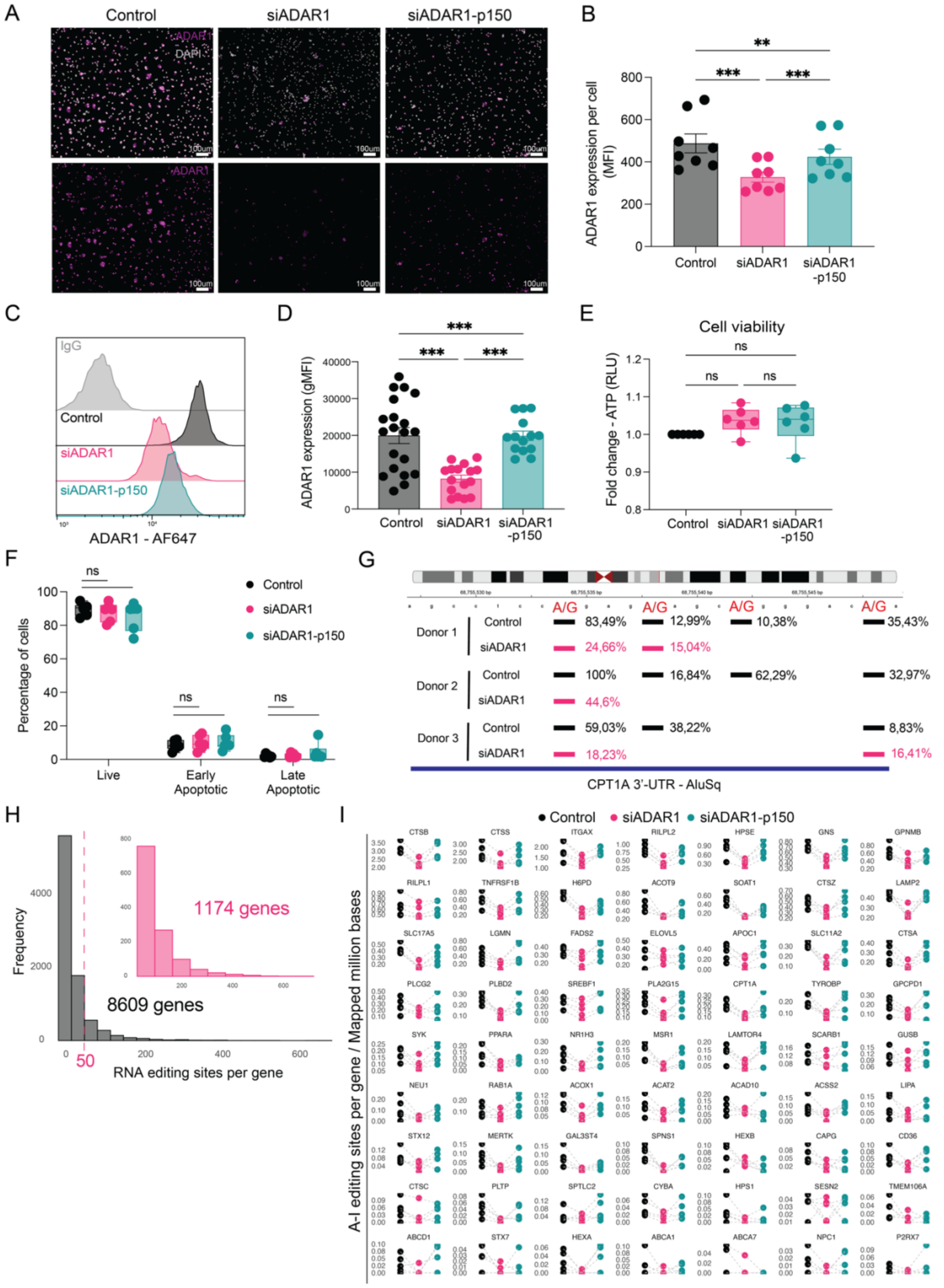

Fig. S3. Effects of ADAR1 silencing on macrophage viability and RNA editing. Human MDMs were transfected with control, siADAR1 or siADAR1-p150. (**A**, **B**) Representative 20x

immunofluorescence images and quantification of ADAR1 in transfected MDMs, stained with ADAR1 (purple) and with DAPI (grey). Scale bar, 100μm. For each donor, MFI was averaged across all detected cells from 4-5 representative images. (**C**, **D**) Representative flow cytometry histograms and quantification of ADAR1 expression (gMFI) in transfected MDMs after 48hrs. (**E**) Box plots showing fold changes in intracellular ATP levels, measured as relative luminescence units (RLU), in transfected MDMs (n = 6). Each biological replicate represents the mean of three technical replicates. (**F**) Box plots showing the cell percentages of Live (Annexin V^-/Propidium $Iodine^-$), Early Apoptotic (Annexin V^+/PI^-) and Late apoptotic (Annexin V^+/PI^+) in transfected MDMs (n = 6). (**G**) Integrative genomics viewer (IGV) browser snapshot of the *CPT1A* 3'-UTR (AluSq, chr11: 68,755,527-68,755,549) showing detected A-to-I RNA editing sites and their editing frequency in control and siADAR1-transfected MDMs (n = 3). (**H**) Histogram displaying the distribution of detected A-to-I RNA editing events per gene (grey). Genes containing ≥50 editing events (pink) prompted for pathway enrichment analysis. (**I**) Paired dot plots displaying the number of detected A-to-I RNA editing sites per genes, normalized to sequencing depth, across 63 genes associated with lysosome function, lipid metabolism, phagocytosis/endocytosis, and TYROBP signalling pathways in transfected MDMs (n = 5). Genes are ranked by total editing frequency from highest to lowest. Data in bar plots are presented as the mean ± SEM. Box-and-whisker plots display min-max range, interquartile range (25th-75th percentiles) and median. Statistical significance was determined by one-way ANOVA (**B**, **E, F**) or mixed-effects analysis (**D**) followed by Tukey's multiple comparisons test. *P < 0.05, **P < 0.01, and ***P < 0.001.

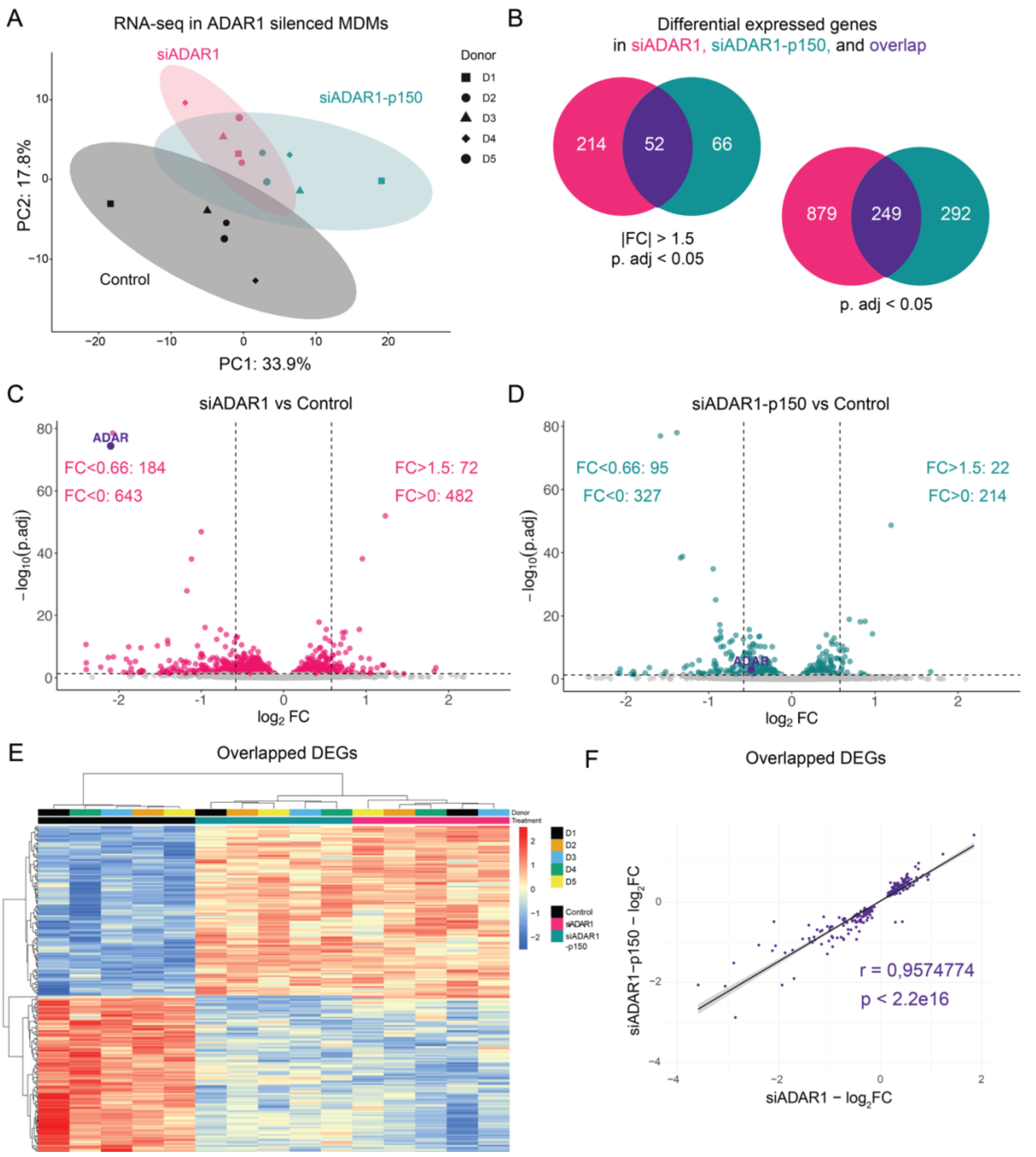

Fig. S4. RNA-sequencing analysis of ADAR1 silencing in human macrophages. (**A**) Principal component analysis (PCA) of gene expression in siADAR1 and siADAR1-p150 relative to control MDMs (n = 5). Data were batch-corrected to account for donor-to-donor variability. (**B**) Venn diagrams showing the number of DEGs with and without fold change (FC) thresholds in siADAR1 and siADAR1-p150 relative to control. (**C, D**) Volcano plots depicting DEGs for siADAR1 (**C**) and siADAR1-p150 (**D**) compared to control (n=5).

Numbers of DEGs passing the indicated FC threshold are indicated for each condition and direction. (**E**) Heatmap showing the normalized counts of overlapping DEGs between siADAR1 and siADAR1-p150 relative to control. Values are shown after donor batch correction. (**F**) Correlation plot of $\log_2$ FC for overlapping DEGs between siADAR1 and siADAR1p-150 relative to control. Pearson correlation coefficient (r) and p-value are indicated. Significance cutoffs of Benjamini-Hochberg (BH)-adjusted p-values < 0.05 were employed for all analyses.

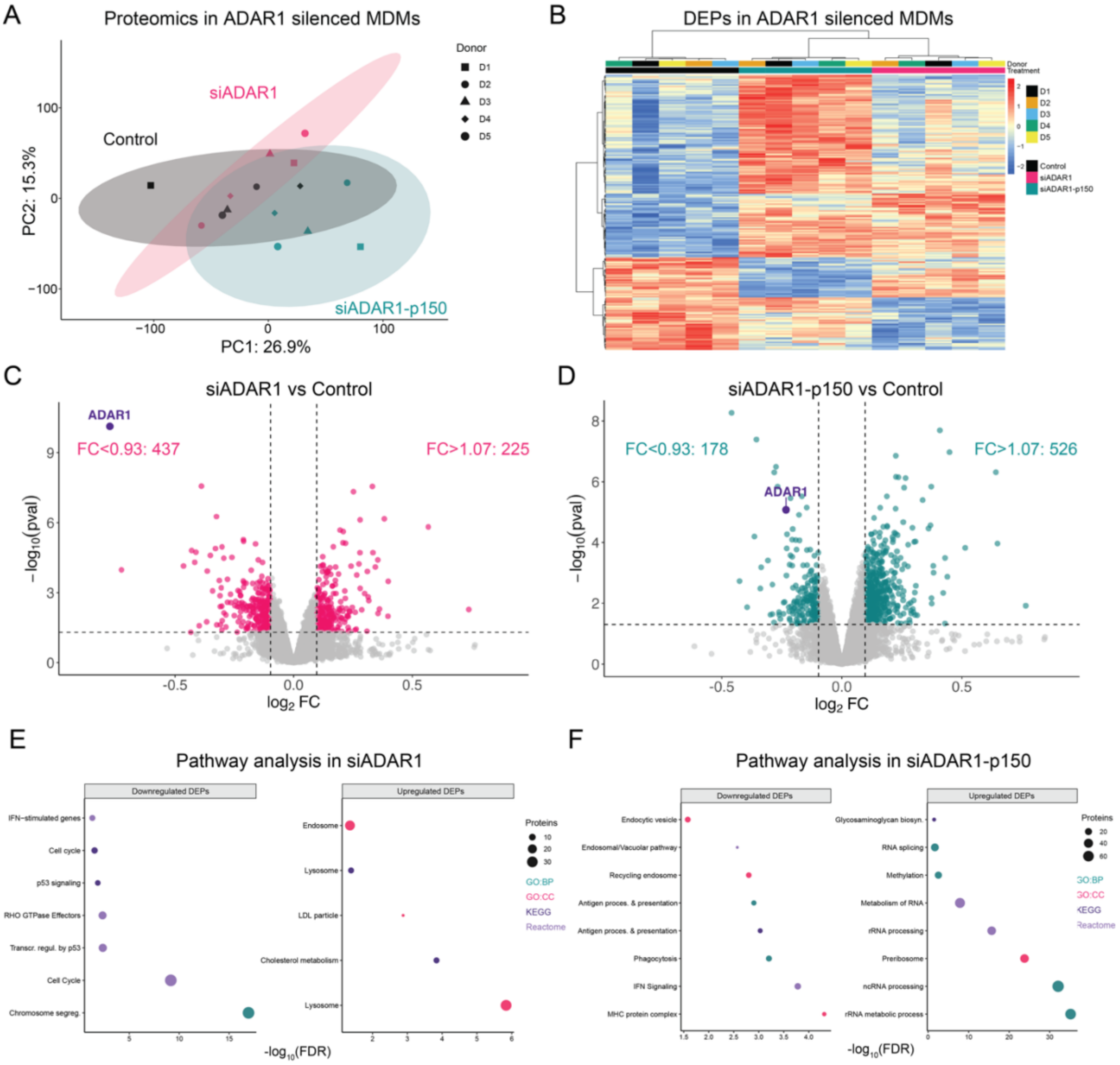

Fig. S5. Global TMT proteomics analysis of ADAR1 silencing in human macrophages. (**A**) Principal component analysis (PCA) of protein expression in siADAR1 and siADAR1p150

relative to control MDMs (n = 5). Data were batch-corrected to account for donor-to-donor variability. (**B**) Heatmap showing normalized log_2 abundances of DEPs between siADAR1 and siADAR1p150 relative to control. Values are shown after donor batch correction. (**C, D**) Volcano plots depicting DEPs for siADAR1 (**C**) and siADAR1-p150 (**D**) relative to control. Numbers of DEPs passing the indicated fold change threshold are indicated for each condition and direction. (**E, F**) Dot plots summarizing overrepresentation analysis of DEPs for siADAR1(**E**) and siADAR1-p150 (**F**) relative to control. Each panel contains separate plots for downregulated and upregulated DEPs, analyzed across indicated functional databases. Significance cutoffs of Benjamini-Hochberg (BH)-adjusted p-values < 0.05 (**B**, **E**, **F**) or p values (**C**, **D**) were employed.

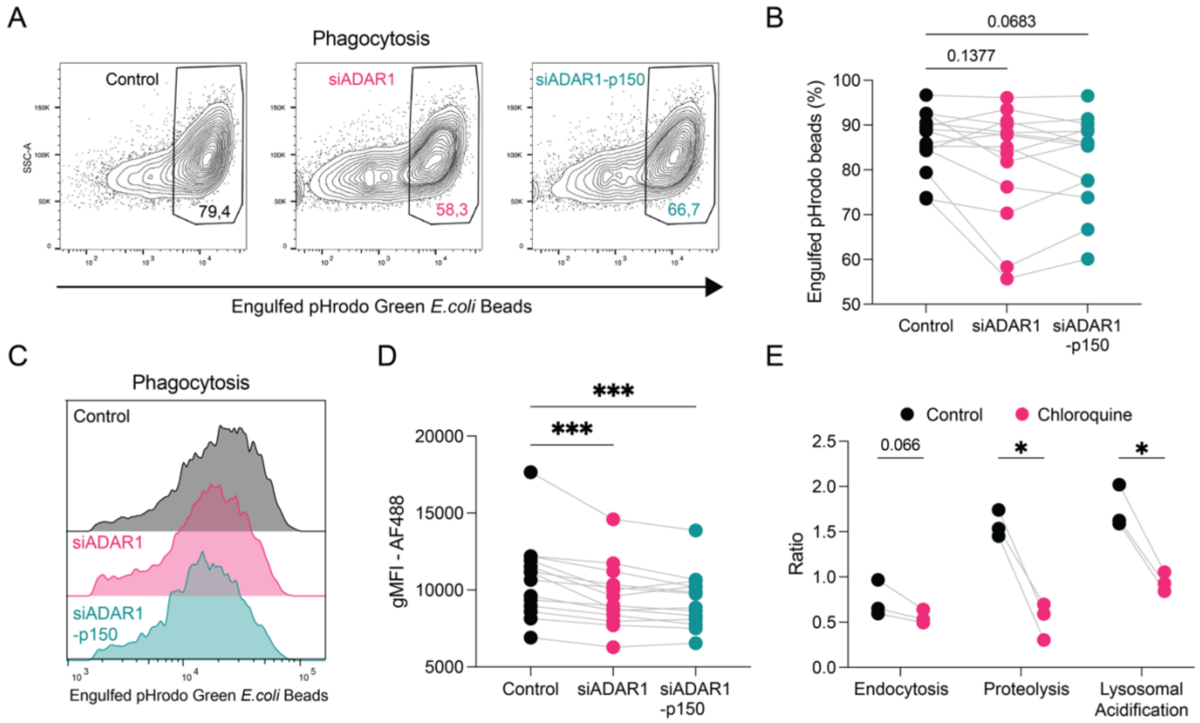

Fig. S6. Effects of ADAR1 silencing on phagocytosis and chloroquine responses in macrophages. (**A**, **B**) Representative flow cytometry plots and frequency of transfected MDMs engulfing pHrodo Green-labeled *E. coli* after 60 minutes incubation. Data are obtained from 4 independent experiments (n = 14). (**C**, **D**) Representative histograms and quantification of

engulfed pHrodo Green-labeled *E. coli* after 60 min incubation from 4 independent experiments (n = 14). (**E**) Paired dot plots showing ratiometric measurements of Endocytosis (pHrodo Dextran Green: Dextran AF647), Proteolysis (DQ BSA-AF647: LysoSensor), and Lysosomal acidification (LysoSensor Green: LysoTracker Red) in control and chloroquine-treated (50 uM, overnight) MDMs (n = 3). Statistical significance was determined by one-way ANOVA followed by Tukey's multiple comparisons test (**B**, **D**) or ratio paired t-test (**E**). *P < 0.05, **P < 0.01, and ***P < 0.001. *P < 0.05, **P < 0.01, and ***P < 0.001.

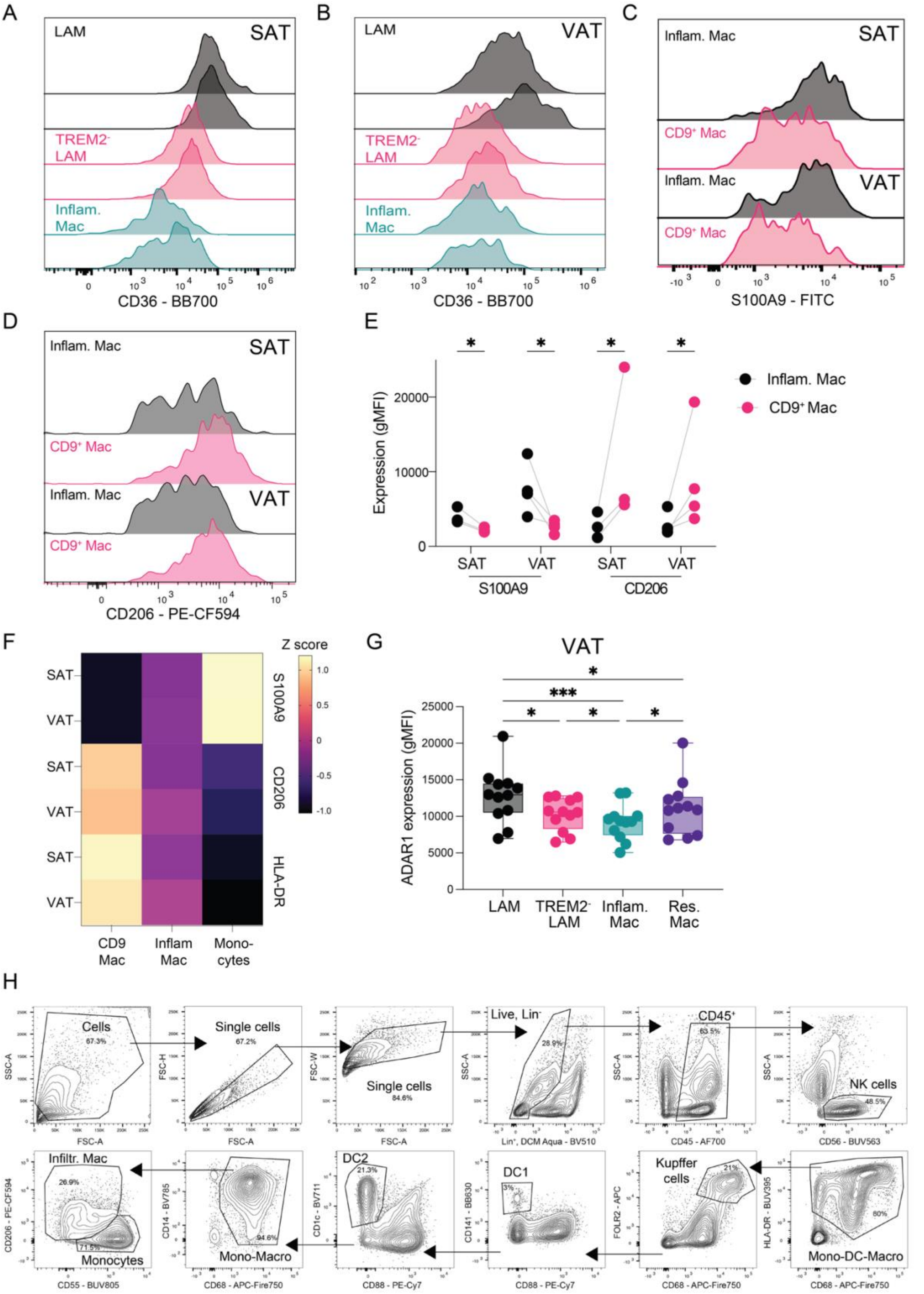

Fig. S7. Delineating macrophage subpopulations in SAT, VAT and liver biopsies. (**A**, **B**) Representative histograms showing CD36 protein expression (gMFI) in infiltrating ATMs

within SVF isolated from SAT and VAT samples from two individual donors. (**C**, **D & E**) Representative histograms and paired dot plots of S100A9 and CD206 expression (gMFI) in infiltrating ATMs within the SVF from SAT (n = 3) and VAT (n = 4) samples. (**F**) Heatmap displaying mean Z-score (gMFI) of S100A9, CD206 and HLA-DR expression in infiltrating ATM subsets and monocytes from SAT (n = 3) and VAT (n = 4) (**G**) Quantification for ADAR1 expression (gMFI) in indicated cell types from SVF cells from VAT biopsies (n = 12). (**H**) Representative flow cytometry gating strategy for NPCs isolated from perfused human livers. Statistical significance was determined by ratio paired t-test (**E**) or one-way ANOVA followed by Tukey's multiple comparisons test (**G**). *P < 0.05, **P < 0.01, and ***P < 0.001.

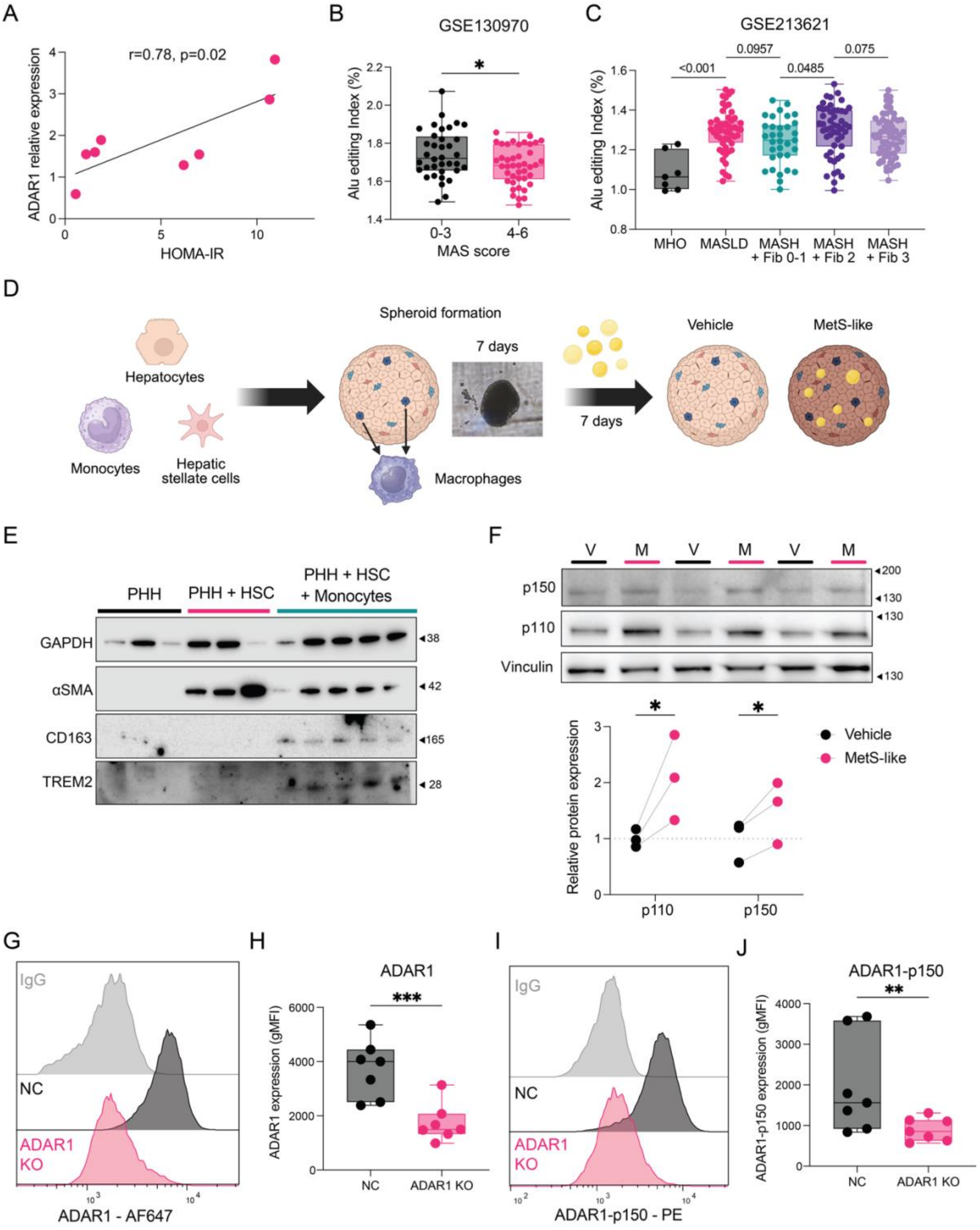

Fig. S8. Regulation of ADAR1 in MASLD, establishment of organotypic spheroid model, and CRISPR KO ADAR1 in human macrophages. (**A**) Correlation plot between relative hepatic ADAR1 protein expression and HOMA-IR in patients with obesity. Pearson correlation coefficient (r) and p-value are indicated. (**B**) Box-and-whisker plots displaying the Alu editing

index in liver biopsies from MASLD (n = 36) and MASH (n = 42) patients. Patients were stratified based on MASLD activity score (MAS score; MASLD = 0-3 and MASH = 4-6). (GSE130970). (**C**) Box-and-whisker plots displaying the Alu editing index in liver biopsies from MHO (n = 7), MASLD (n = 51) and MASH patients stratified by fibrosis stage (Fib 0-1, n = 33; Fib 2, n = 51; Fib 3, n = 64). (GSE213621). (**D**) Schematic illustration of the 3D organotypic hepatic spheroid model. (**E**) Western blot analysis of αSMA, CD163, and TREM2 expression in hepatic spheroids comprised of PHH (n = 3), PHH:HSC (n = 3), and PHH:HSC:Monocytes (n = 5), assessed 7 days post-seeding from two independent experiments. GAPDH was used as the loading control. (**F**) Western blot analysis of ADAR1 protein expression (p110 and p150 isoforms) in hepatic spheroids (PHH: HSC:Monocytes) treated with MetS-like or vehicle for 7 days and summary data for the relative protein expression for both ADAR1 isoforms (n = 3). Vinculin was used as the loading control. (**G-J**) Representative flow cytometry histograms and quantification of total ADAR1 and p150 expression (gMFI) in indicated CRISPR KO MDMs following 7 days of differentiation under MCSF from two independent experiments (n = 7). Box-and-whisker plots display the min-max range, interquartile range (25th-75th percentiles) and median. Statistical significance was determined by Mann-Whitney test (**B, C**) or ratio p t-test (**F, H, J**). *$P < 0.05$, **$P < 0.01$, and ***$P < 0.001$.

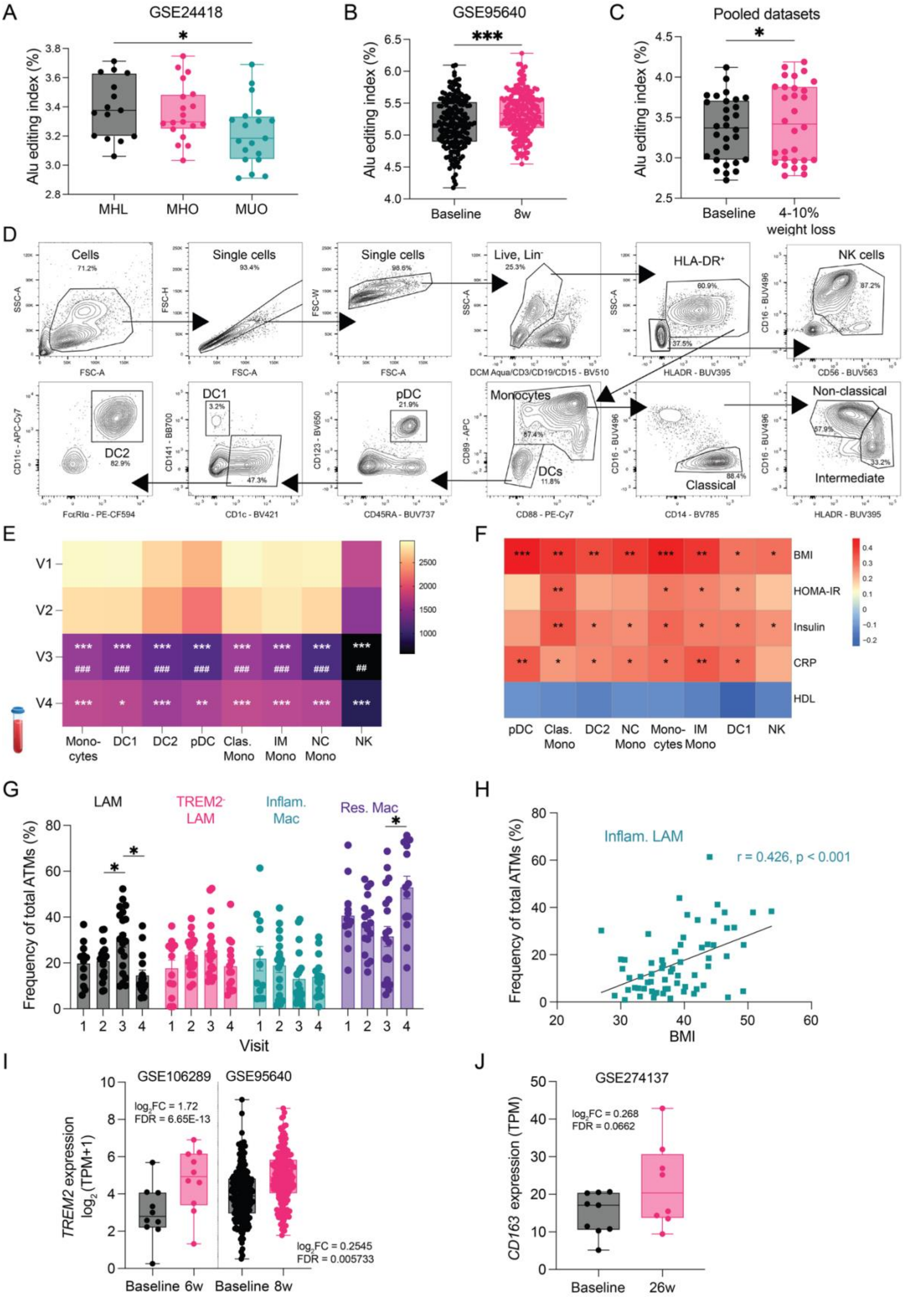
A
GSE24418
Alu editing index (%)
MHL
MHO
MUO
B
GSE95640
Baseline
8w
C
Pooled datasets
Baseline
4-10% weight loss
D
Cells
Single cells
Live, Lin-
HLA-DR+
NK cells
DC1
pDC
Monocytes
DCs
DC2
Classical
Non-classical
Intermediate
E
V1
V2
V3
V4
Mono-cytes
DC1
DC2
pDC
Clas. Mono
IM Mono
NC Mono
NK
F
BMI
HOMA-IR
Insulin
CRP
HDL
G
LAM
TREM2- LAM
Inflam. Mac
Res. Mac
Frequency of total ATMs (%)
Visit
H
Inflam. LAM
r = 0.426, p < 0.001
BMI
I
GSE106289
GSE95640
TREM2 expression log2 (TPM+1)
log2FC = 1.72
FDR = 6.65E-13
log2FC = 0.2545
FDR = 0.005733
Baseline
6w
Baseline
8w
J
GSE274137
CD163 expression (TPM)
log2FC = 0.268
FDR = 0.0662
Baseline
26w

Fig. S9. Regulation of ADAR1 in adipose tissue and peripheral immune cells in response to weight-loss. (**A**) Box-and-whisker plots of Alu editing index in SAT biopsies from MHL (n = 15), MHO (n = 19), and MUO (n = 19) individuals (GSE24418). (**B**, **C**) Box-and-whisker plots of Alu editing index in SAT biopsies in patients undergoing diet-induced weight loss, with (**C**) showing AEI values pooled from three smaller intervention cohorts. (**D**) Representative flow cytometry gating strategy in PBMC subpopulations from patients with obesity in COCKTAIL study. (**E**) Heatmap showing the median ADAR1 expression (gMFI) in indicated PBMC subpopulations from patients with obesity undergoing VLED (n = 7) and bariatric surgery (n = 9) across all visits (COCKTAIL study). *Display the comparison to V1 and $^{\#}$ the comparison to V2. (**F**) Spearman correlation heatmap of ADAR1 expression (gMFI) and indicated clinical and anthropometric parameters in indicated PBMC subpopulations from participants in COCKTAIL study. (**G**) Frequency of ATM subpopulations out of live, $CD45^+$, lin^-, $HLA\text{-}DR^+$, $CD14^+$, $CD88^+$, $CD206^+$ SVF cells from patients with obesity undergoing VLED (n = 7) and bariatric surgery (n = 13) across all visits. (**H**) Correlation plot of Inflam. Mac frequency (%) in total ATM from SAT SVF and BMI from patients with severe obesity undergoing VLED (n = 7) and RYGB surgery (n = 13) across all visits. Spearman correlation coefficient (r) and p-value are indicated. (**I**) Box-and-whisker plots displaying *TREM2* mRNA (log_2(TPM+1)) in SAT biopsies from patients with obesity undergoing 6-week and 8-week caloric diet compared to baseline. (**J**) Box-and-whisker plots displaying *CD163* mRNA (TPM) in SAT biopsies from patients with obesity undergoing 26-week caloric diet compared to baseline. Log_2 Fold change (FC) and FDR values were extracted from the deposited DEG lists in the original studies. Box-and-whisker plots display min-max range, interquartile range (25th-75th percentiles) and median. Data in bar plot are presented as mean ± SEM. Statistical significance was determined by Wilcoxon signed-ranked test (**A**, **B, C**), or mixed-effect

analysis followed by Tukey's multiple comparisons test (**E, G**). *P < 0.05, **P < 0.01, and ***P < 0.001.

Table S1. Cross-referenced annotation of CD206$^+$ adipose tissue macrophages (ATM) subsets from single-cell and single nucleus RNA-seq studies.

Assigned subset name	Flow Cytometry Markers	Annotation markers for human ATM clusters per study			
		**Massier et al. 2023 (SAT, VAT)**	**Boesch et al. 2024 (VAT)**	**Hildreth et al. 2021 (SAT)**	**Vijay et al. 2020 (SAT)**
LAM	CD11c, CD9, TREM2, CD36^{high}	myC02_LAM - *TREM2*, *CD9*, *CD36*	MMac – *TREM2*	LAM - CD11c, *CD36*, *CD9*, *TREM2*	IS2 – *CD36*, *CD9*, *TREM2*
TREM2$^-$ LAM	CD11c, CD9		TransMac – *CD9*		
Inflam. Mac	CD11c, CD206^{mid}, S100A9		Pre. Inflam. Mac – *S100A9*	IM - CD11c, CD206^{mid}, *S100A9*	
Res Vac Mac	CD163^{high}	myC07 – *CD163*	ResVAM – *CD163*		

Abbreviations: LAM: Lipid-associated macrophages, MMac: Metabolically active macrophages, TransMac: Transitional macrophages, Inflam. Mac/IM: Inflammatory macrophages, Res Vac Mac/ResVAM: Resident vasculature-associated macrophages

Table S2. Patient list of liver NPC samples used for flow cytometry.

Patient	Sex	Age	Cause of Death	Type of donation	Panel	Figure
LF1	F	48	Trauma	Donor	Liver panel 1	Figure 1E, 1F and S1C
LF2	F	50	Trauma head injury	Donor	Liver panel 1	
LF3	F	68	Cerebral haemorrhage	Donor	Liver panel 1	
LF4	F	73	Stroke	Donor	Liver panel 2	
LF5	F	76	Intracranial haemorrhage	Donor	Liver panel 2	
LF6	M	65	Cerebral infarction	Donor	Liver panel 1	
LF7	M	72	SDH	Donor	Liver panel 2	
LF8	M	55	Cardiac arrest	Donor	Liver panel 1	
LF9	M	76	Intracranial haemorrhage	Donor	Liver panel 2	

LF10	M	65	Trauma	Donor	Liver panel 2	
LF11	M	82	Unknown	Resection	Liver panel 3	Figure 6F-H
LF12	M	54	Unknown	Resection		
LF13	F	53	CRC	Resection		
LF14	M	71	CRC	Resection		
LF15	M	65	CRC	Resection		
LF16	F	59	Mucinous cystic neoplasm	Resection		
LF17	M	73	SAH	Donor		
LF18	M	23	Cardiac arrest	Donor		
LF19	Unknown	Unknown	Unknown	Donor		
LF20	M	55	Cardiac arrest	Donor		
LF21	M	45	CRC	Resection		

Abbreviations: F: Female, M: Male, SDH: Subdural hemangioma, CRC: Colorectal Cancer, SAH: Subarachnoid hemorrhage

Table S3. Patient list of liver FFPE biopsies used for immunofluorescence.

**Patient**	**MASLD**	Early MASH	Late MASH
Patients	5	5	6
Sex (F/M)	3/2	1/4	3/3
Age (years)	51 (43-72)	45 (31-65)	68 (30-70)
MAS (0-8)	2.4 ± 1.14	4.5 ± 0.58	5.67 ± 1.21
Fibrosis stage	0.4 ± 0.55	1.2 ± 0.45	2.83 ± 1.17

Abbreviations: F: Female, M: Male, MAS score**:** MASLD Score

Table S4. Clinical information of liver biopsies from normal weight individuals used for Western blot analysis.

**Patient**	**Age**	**Sex**	**BMI**	**Cause of Death**
LW1	68	M	24.9	ICH
LW2	68	M	24.9	ICH
LW3	63	M	23.5	SDH
LW4	59	M	21.3	Cardiac arrest

Abbreviations: M: Male, ICH: Intracranial hemorrage, SDH: subdural hemangioma

Table S5. Clinical information of liver biopsies from patients with obesity used for Western blot analysis.

Method	Western Blot
Patients	8
Sex (F/M)	0 / 8
Age (years)	41 (25–44)
BMI (kg/m²)	35.8 (35.8–40.8)
HOMA-IR	4.03 (0.56–10.94)
Diabetes (Y/N)	0 / 8

Abbreviations: F: Female, M: Male, Y: Yes, N: No

Table S6. Clinical information of liver, VAT and SAT biopsies from patients with obesity used for flow cytometry and immunofluorescence analyses.

Method	Flow cytometry	Immunofluorescence
Patients	14	6
Sex (F/M)	11 / 3	6 / 0
Age (years)	45 (26–56)	33 (27–39)
BMI (kg/m²)	36.7 (35.1–41.5)	38.9 (37.6 –40.8)
HOMA-IR	4.64 (2.22–10.83)	2.98 (2.44–10.4)
Diabetes (Y/N/U)	3 / 10 / 1	0 / 6 / 0

Abbreviations: F: Female, M: Male, BMI: Body Mass Index, HOMA-IR: Homeostatic Model Assessment of Insulin Resistance. Y: Yes, N: No, U: Unknown

Table S7. Clinical information and sample inclusion/exclusion of SAT SVF samples used for flow cytometry in the COCKTAIL study, grouped by visit.

	Visit 1	Visit 2	Visit 3	Visit 4
VLED / RYGB	6 / 13	7 / 12	7 / 13	7 / 9
Sex (F/M)	15 / 4	16 / 3	16 / 4	14 / 2
Age (years)	48 (24–68)	48 (24–68)	48 (24–68)	48 (24–69)
Weight (kg)	117.2 (83.3–161.5)	109.8 (79.2–151.7)	104 (71.6–148.9)	97.3 (71.8–135)
BMI (kg/m²)	41.7 (31.4–53.7)	39.2 (29.8–50.8)	36.6 (26.9–46.4)	34.3 (30.3–46.7)
HOMA-IR	2.2 (0.8–7.2)	1.4 (0.4–2.6)	1.2 (0.4–5.3)	0.7 (0.4–1.4)
Insulin (pmol/L)	114 (56–317.5)	89 (35–161)	81.5 (36–309)	59.5 (35–102)
Glucose (mmol/L)	0.4 (0.2–0.8)	0.3 (0.2–0.6)	0.3 (0.2–0.4)	0.3 (0.2–0.4)
CRP (mg/L)	6.5 (0.5–33)	3.6 (0.6–24)	2.2 (0.4–26)	3.1 (0.8–15)
Cholesterol (mmol/L)	4.8 (3.3–6.7)	4.1 (2.2–7.7)	3.5 (1.9–6.5)	4 (3.2–6)
HDL (mmol/L)	1 (0.7–1.6)	1 (0.6–1.5)	1 (0.5–1.4)	1.2 (0.7–1.7)
LDL (mmol/L)	2.8 (1.4–3.6)	2.6 (0.6–5.7)	2 (0.6–4.1)	2.3 (1.8–4)
Triglycerides (mmol/L)	1.5 (0.7–11.4)	1.2 (0.6–2.4)	1 (0.6–3.2)	1 (0.6–2.4)
ALAT (U/L)	25.5 (9–67)	32 (10–116)	32.5 (9–188)	19 (9–61)

Abbreviations: F: Female, M: Male, VLED: Very Low-Energy Diet, RYGB: Roux-en-Y Gastric Bypass, BMI: Body Mass Index, HOMA-IR: Homeostatic Model Assessment of Insulin Resistance, CRP: C-Reactive Protein, HDL: High-Density Lipoprotein cholesterol, LDL: Low-Density Lipoprotein cholesterol, ALAT: Alanine Aminotransferase

Samples from four participants in the RYGB group were not collected at V4. In addition, one V2 sample from the RYGB group was excluded from analysis due to incomplete anthropometric and clinical data for that visit. One V1 sample from the VLED group was also excluded because the participant did not adhere to the LED protocol and failed to achieve the expected weight reduction between V1 and V2.

Table S8. Clinical information and sample exclusion of PBMCs used for flow cytometry in the COCKTAIL study, grouped by visit.

	Visit 1	Visit 2	Visit 3	Visit 4
VLED / RYGB	7 / 9	6 / 9	7 / 9	7 / 9
Sex (F/M)	14 / 2	13 / 2	14 / 2	14 / 2
Age (years)	47.5 (24–68)	47.5 (24–68)	47.5 (24–69)	48 (24–69)
Weight (kg)	117.2 (91.1–152.8)	109.6 (87–145.2)	104 (78.5–148.9)	97.2 (71.8–135)
BMI (kg/m²)	41.7 (34.9–53.7)	38.9 (33–50.8)	36.6 (30–46.4)	34.3 (30.3–46.7)
HOMA-IR	1.9 (0.8–7.2)	1.3 (0.4–2.2)	1 (0.4–5.3)	0.7 (0.4–1.4)
Insulin (pmol/L)	101.5 (67–317.5)	89 (35–161)	79.5 (38–309)	60.2 (35–102)
Glucose (mmol/L)	0.3 (0.2–0.6)	0.3 (0.2–0.5)	0.3 (0.2–0.4)	0.3 (0.2–0.4)
CRP (mg/L)	8 (0.6–33)	4.3 (0.6–24)	2.7 (0.4–26)	2.8 (0.8–15)
Cholesterol (mmol/L)	4.8 (4.1–6.7)	4 (3.1–7.7)	3.6 (2.5–6.5)	4.2 (3.2–6)
HDL (mmol/L)	1.2 (0.8–1.8)	1 (0.8–1.5)	1 (0.5–1.4)	1.2 (0.7–1.7)
LDL (mmol/L)	2.8 (2.5–3.6)	2.5 (1.5–5.7)	2 (0.9–4.1)	2.4 (1.8–4)
Triglycerides (mmol/L)	1.2 (0.7–11.4)	1.1 (0.6–2.4)	0.9 (0.6–3.2)	1 (0.6–2.4)
ALAT (U/L)	22 (9–67)	32 (12–57)	32 (9–66)	18.5 (9–61)

Abbreviations: F: Female, M: Male, VLED: Very Low-Energy Diet, RYGB: Roux-en-Y Gastric Bypass, BMI: Body Mass Index, HOMA-IR: Homeostatic Model Assessment of Insulin Resistance, CRP: C-Reactive Protein, HDL: High-Density Lipoprotein cholesterol, LDL: Low-Density Lipoprotein cholesterol, ALAT: Alanine Aminotransferase

One V2 sample from the VLED group was also excluded because of its extremely low quality upon thawing.

Table S9. List of siRNA & crRNAs used in human MDMs.

Target	Product	Sense / Anti-sense
Control	Silencer Select Negative Control No 1	
ADAR1	Silencer Select Pre-Designed siRNA, ADAR1, S1007	
ADAR1-p150	Silencer Select Custom-made	GCCUCGCGGGCGCAAUGAATT / UUCAUUGCGCCCGCGAGGCAT
cr-NTC1	Alt-R™ CRISPR-Cas9 crRNA	CGTTAATCGCGTATAATACG
cr-NTC2	Alt-R™ CRISPR-Cas9 crRNA	CATATTGCGCGTATAGTCGC
cr-AltTSS_ADAR1_1	Alt-R™ CRISPR-Cas9 crRNA	GACCATTGATTCCCGACTGA
cr-AltTSS_ADAR1_2	Alt-R™ CRISPR-Cas9 crRNA	ATGACTAAGGAGTTCAATCA
cr-ADAR_1	Alt-R™ CRISPR-Cas9 crRNA	AAGGGACAACCCCTCCCATA
cr-ADAR_2	Alt-R™ CRISPR-Cas9 crRNA	TTATATCTCGGGCCTTGGTA

Table S10. Flow cytometry panel for PBMCs from healthy human buffy coat donors.

Laser	Filter	Fluorochrome	Antigen / AB	Manufacturer	Clone	Dilution
355	379/28	BUV395	HLA-DR	BD Biosciences	G46-6	100
	515/30	BUV496	CD19	BD Biosciences	SJ25C1	50
	580/20	BUV563	CD8	BD Biosciences	RPA-T8	100
	670/25	BUV661	TCRva7.2	BD Biosciences	OF-5A12	100
	735/50	BUV737	CD45RA	BD Biosciences	HI100	300
	810/40	BUV805	CD3	BD Biosciences	SK7	100
405	450/50	BV421	CD1c	BioLegend	L161	50
	525/50		Zombie Aqua	BioLegend		1000
	605/40	BV605	FcεR1α	BD Biosciences	AER-37	50
	677/20	BV650	CD161	BD Biosciences	DX12	25
	710/50	BV711	CD123	BD Biosciences	9F5	200
	810/40	BV785	CD16	BD Biosciences	3G8	200
488	530/30	AF488	ADAR1*	Cell Signaling		50
	710/50	BB700	CD141	BD Biosciences	1A4	200
561	586/15	PE	FoxP3*	BioLegend	206D	50
	610/20	PE-Dazzle594	CCR7	BioLegend	G043H7	100
	670/30	PE-Cy5	CD4	BioLegend	OKT4	300
	780/60	PE-Cy7	CD56	BD Biosciences	NCAM16.2	150
637	670/30	AF647	CD14	BioLegend	M5E2	100
	730/45	AF700	CD25	BD Biosciences	M-A251	25
	780/60	APC-Cy7	CD11c	BioLegend	BU15	150

Comment: This panel is used in Figure 1A, B and S1A and B

*Intracellular staining

Table S11. Flow cytometry panels for liver NPCs isolated from donor livers rejected for transplantation.

Laser	Filter	Fluorochrome	Antigen / AB	Manufacturer	Clone	Dilution	Panel
355	379/28	BUV395	CD38	BD Biosciences	HB7	100	1 & 2
	515/30	BUV496	CD16	BD Biosciences	3G8	200	1 & 2
	580/20	BUV563	CD56	BD Biosciences	NCAM16.2	200	1 & 2
	605/20	BUV615	FcεR1α	BD Biosciences	AER-37	50	1 & 2
	670/25	BUV661	CD86	BD Biosciences	L293	50	1 & 2
	735/50	BUV737	CCR2	BD Biosciences	LS 132.1D9	200	1 & 2
	810/40	BUV805	CD55	BD Biosciences	IA10	100	1 & 2
405	450/50	BV421	CD206	BD Biosciences	19.2	50	1 & 2
	525/50	BV510	CD3	BD Biosciences	UCHT1	50	1 & 2
		BV510	CD19	BD Biosciences	SJ25C1	100	1 & 2
		V500	CD15	BD Biosciences	HI98	50	1 & 2
			Zombie Aqua	BioLegend		500	1 & 2
	677/20	BV650	CD44	BioLegend	IM7	400	1 & 2
	710/50	BV711	CD123	BD Biosciences	9F5	200	1 & 2
	750/30	BV750	PDL1	BD Biosciences	MIH1	200	1 & 2
488	530/30	AF488	ADAR1*	Cell Signaling		50	1 & 2
	610/20	BB630	CD141	BD Biosciences	1A4	25	1 & 2
	710/50	BB700	CD1c	BD Biosciences	F10 21A3	100	1 & 2
561	586/15	PE	CD88	BioLegend	S5/1	400	1 & 2
	610/20	PE-CF594	IL6R	BD Biosciences	M5	25	1 & 2
	670/30	PE-Cy5	CD14	eBioscience	61D3	100	1 & 2
	780/60	PE-Cy7	CD68*	BioLegend	Y1/82A	200	1 & 2
637	670/30	AF647 / APC	CD163 / FORL2	BD Biosciences	GHI/61 / 94b	50 / 50	1 / 2
	730/45	AF700	CD45	BioLegend	HI30	400	1 & 2
	780/60	APC- Fire 750	HLA-DR	BioLegend	L243	50	1 & 2

Comment: Panel 1 and 2 are used for Figure 1E, F and S1C. The main difference for the panels is the use of CD163 or FOLR2 in the Red channel at 670/30.

*Intracellular staining

Table S12. Flow cytometry panel for liver NPCs isolated from donor livers rejected for transplantation or from patients undergoing resection.

Laser	Filter	Fluorochrome	Antigen / AB	Manufacturer	Clone	Dilution
355	379/28	BUV395	HLA-DR	BD Biosciences	G46-6	100
	515/30	BUV496	CD16	BD Biosciences	3G8	200
	580/20	BUV563	CD56	BD Biosciences	NCAM16.2	200
	605/20	BUV615	FcεR1α	BD Biosciences	AER-37	50
	735/50	BUV737	CCR2	BD Biosciences	LS 132.1D9	200
	810/40	BUV805	CD55	BD Biosciences	IA10	100
405	450/50	BV421	CD163	BioLegend	GHI/61	50
	525/50	BV510	CD3	BD Biosciences	UCHT1	50
		BV510	CD19	BD Biosciences	SJ25C1	100
		BV510	CD123	BioLegend	6H6	50
		V500	CD15	BD Biosciences	HI98	50
			Zombie Aqua	BioLegend		500
	605/40	BV605	CD31	BD Biosciences	WM59	50
	710/50	BV711	CD1c	BioLegend	L161	50
	810/40	BV785	CD14	BioLegend	M5E2	100
488	530/30	AF488	ADAR1*	Cell Signaling		50
	610/20	BB630	CD141	BD Biosciences	1A4	25
	710/50	BB700	CD26	BD Biosciences	L272	100
561	586/15	PE	S100A9*	BioLegend	MRP 1H9	250
	610/20	PE-CF594	CD206	BD Biosciences	19.2	50
	780/60	PE-Cy7	CD88	BioLegend	S5/1	100
637	670/30	APC	FOLR2	BioLegend	94b	50
	730/45	AF700	CD45	BioLegend	HI30	400
	780/60	APC-Fire750	CD68*	BioLegend	Y1/82A	100

Comment: This panel is used in Figure 6F-H, Fig S7G

*Intracellular staining

Table S13. Flow cytometry panel for SAT and VAT SVF cells from patients with obesity and patients participating in the COCKTAIL study.

Laser	Filter	Fluorochrome	Antigen / AB	Manufacturer	Clone	Dilution
355	379/28	BUV395	HLA-DR	BD Biosciences	G46-6	100
	515/30	BUV496	CD16	BD Biosciences	3G8	200
	580/20	BUV563	CD56	BD Biosciences	NCAM16.2	200
	605/20	BUV615	CD9	BD Biosciences	M-L13	50
	735/50	BUV737	CCR2	BD Biosciences	LS 132.1D9	200
	810/40	BUV805	CD55	BD Biosciences	IA10	100
405	450/50	BV421	CD163	BioLegend	GHI/61	50
	525/50	BV510	CD3	BD Biosciences	UCHT1	50
		BV510	CD19	BD Biosciences	SJ25C1	100
		BV510	CD123	BioLegend	6H6	50
		V500	CD15	BD Biosciences	HI98	50
			Zombie Aqua	BioLegend		500
	677/20	BV650	CD34	BioLegend	561	100
	710/50	BV711	CD1c	BioLegend	L161	50
	810/40	BV785	CD14	BioLegend	M5E2	100
488	530/30	AF488	ADAR1*	Cell Signaling		50
	610/20	BB630	CD141	BD Biosciences	1A4	25
	710/50	BB700	CD36	BD Biosciences	FA6-152	100
561	586/15	PE	ADAR1p150*	Abcam	EPR7033	50
	610/20	PE-CF594	CD206	BD Biosciences	19.2	25
	780/60	PE-Cy7	CD88	BioLegend	S5/1	100
637	670/30	APC	TREM2	R&D Systems	237920	10
	730/45	AF700	CD45	BioLegend	HI30	400
	780/60	APC-Cy7	CD11c	BioLegend	BU15	100

Comment: This panel is used in Fig. 1G-I, S1D, 6A-E, S7A, B, F, Figure 8E & F, S9G & H

*Intracellular staining

Table S14. Complementary flow cytometry panel for SAT and VAT SVF cells from patients with obesity.

Laser	Filter	Fluorochrome	Antigen / AB	Manufacturer	Clone	Dilution
355	379/28	BUV395	HLA-DR	BD Biosciences	G46-6	100
	515/30	BUV496	CD16	BD Biosciences	3G8	200
	580/20	BUV563	CD56	BD Biosciences	NCAM16.2	200
	605/20	BUV661	FcεR1α	BD Biosciences	AER-37	50
	735/50	BUV737	CCR2	BD Biosciences	LS 132.1D9	200
	810/40	BUV805	CD55	BD Biosciences	IA10	100
405	450/50	BV421	CD163	BioLegend	GHI/61	50
	525/50	BV510	CD3	BD Biosciences	UCHT1	50
		BV510	CD19	BD Biosciences	SJ25C1	100
		V500	CD15	BD Biosciences	HI98	50
			Zombie Aqua	BioLegend		500
	677/20	BV650	CD123	BioLegend	6H6	100
	710/50	BV711	CD1c	BioLegend	L161	50
	810/40	BV785	CD14	BioLegend	M5E2	100
488	530/30	FITC	S100A9*	BioLegend	MRP 1H9	200
	610/20	BB630	CD141	BD Biosciences	1A4	25
	710/50	PerCP-Cy 5.5	CD88	BioLegend	S5/1	100
561	586/15	PE	CD9	Miltenyi	SN4 C3-3A2	100
	610/20	PE-CF594	CD206	BD Biosciences	19.2	25
	780/60	PE-Cy7	CD200R1	BioLegend	OX-108	100
637	670/30	AF647	ADAR1*	Cell Signaling		50
	730/45	AF700	CD45	BioLegend	HI30	400
	780/60	APC-Cy7	CD11c	BioLegend	BU15	100

Comment: This panel is used in Figure S7C-E

*Intracellular staining

Table S15. Flow cytometry panel for PBMCs from patients participating in the COCKTAIL study.

Laser	Filter	Fluorochrome	Antigen / AB	Manufacturer	Clone	Dilution
355	379/28	BUV395	HLA-DR	BD Biosciences	G46-6	100
	515/30	BUV496	CD16	BD Biosciences	3G8	200
	580/20	BUV563	CD56	BD Biosciences	NCAM16.2	200
	735/50	BUV737	CD45RA	BD Biosciences	HI100	200
	810/40	BUV805	CD55	BD Biosciences	IA10	100
405	450/50	BV421	CD1c	BioLegend	L161	50
	525/50	BV510	CD3	BD Biosciences	UCHT1	50
		BV510	CD19	BD Biosciences	SJ25C1	100
		V500	CD15	BD Biosciences	HI98	50
			Zombie Aqua	BioLegend		1000
	677/20	BV650	CD123	BioLegend	6H6	50
	710/50	BV711	CD5	BD Biosciences	UCHT2	100
	810/40	BV785	CD14	BioLegend	M5E2	100
488	530/30	AF488	ADAR1*	Cell Signaling		50
	710/50	BB700	CD141	BD Biosciences	1A4	100
561	586/15	PE	ADAR1p150*	Abcam	EPR7033	50
	610/20	PE-CF594	FcεR1α	BioLegend	AER-37	100
	780/60	PE-Cy7	CD88	BioLegend	S5/1	50
637	670/30	APC	CD89	BioLegend	A59	50
	730/45	AF700	CD14	BD Biosciences	M5E2	50
	780/60	APC-Cy7	CD11c	BioLegend	BU15	100

Comment: This panel is used in Figure S9D, E, and F

*Intracellular staining

Table S16. List of antibodies used for flow cytometry in human MDMs.

Target	Fluorochrome	Manufacturer	Clone	Localization	Dilution
MSR1	BUV395	BD Biosciences	U23-56	Extracellular	50
CD36	BV421	BioLegend	5-271	Extracellular	150
SYK	PE	BioLegend	4D10.2	Intracellular	100
LAMP2	AF594	BioLegend	4HB4	Intracellular	200
SCARB1	APC	BioLegend	m1B9	Extracellular	50
MERTK	AF647	BioLegend	590H11G1E3	Extracellular	100
DAP12	AF647	BD Biosciences	406288	Extracellular	50
CD11c	APC-Cy7	BioLegend	BU15	Extracellular	150
Ki-67	BV421	BioLegend	Ki-67	Intracellular	100
ADAR1-p150	PE	Abcam	EPR7033	Intracellular	50
ADAR1	AF488	Cell Signaling	E6X9R	Intracellular	50
ADAR1	AF647	Cell Signaling	E6X9R	Intracellular	50
IgG	PE	Abcam	EPR25A	Intracellular	50
IgG	AF488	Cell Signaling	DA1E	Intracellular	200
IgG	AF647	Cell Signaling	DA1E	Intracellular	200

Table S17. List of antibodies used for Western blot, IF and IHC.

Target	Manufacturer	Product code	Assay	Dilution	Type of Ab
ADAR1	Cell Signaling	81284S	WB	1:1000	Primary
α-SMA	Sigma	A2547	WB	1:2000	Primary
CD163	BioRad	MCA1853	WB	1:1000	Primary
GAPDH	Invitrogen	PA1-987	WB	1:2000	Primary
Vinculin	Cell Signaling	13901S	WB	1:1000	Primary
α-tubulin	Cell Signaling	2144S	WB	1:1000	Primary
β-actin	Abcam	ab179467	WB	1:5000	Primary
CPT1A	Cell Signaling	12252S	WB	1:1000	Primary
TREM2	Cell Signaling	D8I4C	WB	1:1000	Primary
CD68	Abcam	ab199000	IHC	1:50	Primary
ADAR1	Atlas	HPA003890	ICC	1:200	Primary
ADAR1	Abcam	ab226188	IHC	1:50	Primary
ADAR1 - AF647	Cell Signaling	99623S	ICC	1:100	Conjugated
Goat Anti-Rabbit H&L (HRP)	Abcam	ab6721	WB	1:1000	Secondary
Polyclonal Goat Anti-Mouse HRP	Agilent	P044701-2	WB	1:1000	Secondary
Goat Anti-Rabbit IgG, AF555	Invitrogen	A-21428	IHC	1:500	Secondary
Goat Anti-Mouse IgG1 AF647	Invitrogen	A-21240	IHC	1:500	Secondary

Table S18. List of primers used for qPCR.

Gene	Forward	Reverse	Product (bp)
ADAR	CGCCCTCTTTGACAAGTCCT	ATGGTACGGAGTCTCTCCCC	195
TBP	AGCGCAAGGGTTTCTGGTTT	CTGAATAGGCTGTGGGGTCA	173
